# Virological, immunological and pathological findings of transplacentally transmitted bluetongue virus serotype 1 in IFNAR1-blocked mice during early and mid gestation

**DOI:** 10.1038/s41598-020-58268-0

**Published:** 2020-02-07

**Authors:** M. Saminathan, K. P. Singh, S. Vineetha, Madhulina Maity, S. K. Biswas, G. B. Manjunathareddy, H. C. Chauhan, A. A. P. Milton, M. A. Ramakrishnan, Sushila Maan, N. S. Maan, D. Hemadri, B. S. Chandel, V. K. Gupta, P. P. C. Mertens

**Affiliations:** 1 Division of Pathology, ICAR-Indian Veterinary Research Institute (ICAR-IVRI), Izatnagar, Bareilly, 243122 Uttar Pradesh India; 2 Centre for Animal Disease Research and Diagnosis (CADRAD), ICAR-IVRI, Izatnagar, Bareilly, 243122 Uttar Pradesh India; 3 Division of Virology, ICAR-IVRI, Mukteswar Campus, Nainital, 263138 Uttarakhand India; 4 ICAR-National Institute of Veterinary Epidemiology and Disease Informatics, Bengaluru, 560064 Karnataka India; 5 Department of Veterinary Microbiology, College of Veterinary Science and Animal Husbandry, Sardarkrushinagar Dantiwada Agricultural University, Sardarkrushinagar, 385506 Gujarat India; 6 Division of Animal Health, ICAR-RC for NEH Region, Umiam, Barapani, 793103 Meghalaya India; 7 College of Veterinary Sciences, LLR University of Veterinary and Animal Sciences, Hisar, 125 004 Haryana India; 8 School of Veterinary Medicine and Science, University of Nottingham, Sutton Bonnington, Leicestershire UK

**Keywords:** Viral pathogenesis, Viral infection

## Abstract

Transplacental transmission (TPT) of wild-type Indian BTV-1 had never been experimentally proved. This study was first time investigated TPT of Indian BTV-1 (isolated from aborted and stillborn goat fetal spleens). The sequential pathology, virological and immune cell kinetics (CD4^+^, CD8^+^ T-lymphocytes and NK cells in spleen and PBMCs), and apoptosis in IFNAR1-blocked pregnant mice during early (infected on 1 GD) and mid (infected on 8 GD) gestation have been studied. There was higher rate of TPT during mid stage (71.43%) than early (57.14%) stage. In early stage reduced implantation sites, early embryonic deaths, abortions, and necro-haemorrhagic lesions had observed. Mid stage, congenital defects and neurological lesions in foetuses like haemorrhages, diffuse cerebral edema, necrotizing encephalitis and decreased bone size (Alizarin red staining) were noticed. BTV-1 antigen was first time demonstrable in cells of mesometrium, decidua of embryos, placenta, uterus, ovary, and brain of foetuses by immunohistochemistry and quantified by real-time qRT-PCR. BTV-inoculated mice were seroconverted by 7 and 5 dpi, and reached peak levels by 15 and 9 dpi in early and mid gestation, respectively. CD4^+^ and CD8^+^ cells were significantly decreased (increased ratio) on 7 dpi but subsequently increased on 15 dpi in early gestation. In mid gestation, increased CD8^+^ cells (decreased ratio) were observed. Apoptotic cells in PBMCs and tissues increased during peak viral load. This first time TPT of wild-type Indian BTV-1 deserves to be reported for implementation of control strategies. This model will be very suitable for further research into mechanisms of TPT, overwintering, and vaccination strategies.

## Introduction

Bluetongue (BT) is a non-contagious, insect-borne (mainly biting midges of Culicoides spp.) disease of domestic (primarily sheep) and wild ruminants, caused by bluetongue virus (BTV). BTV is the prototype member of the genus *Orbivirus* in the family *Reoviridae*^1^. Most prominent clinical signs of BT are usually seen in sheep. Subclinical or mild signs are seen in cattle and goats, until the emergence of BTV-1 and BTV-8 in Northern Europe^2,3^. BT is a notifiable disease, listed by World Organization for Animal Health (OIE) and causes huge socio-economic losses and hindrance to the international trade of animals and animal products. BT in sheep causes severe haemorrhagic syndrome characterized by fever, oedema, haemorrhages, dyspnoea, mucosal erosions and ulcerations, and coronitis^3^. BTV infection in pregnant animals has led to abortions, congenital deformities and cerebral malformations such as hydranencephaly, and also the birth of viraemic calves^4–7^.

Till date, 27 distinct BTV serotypes have been described. Between the serotypes, large genetic and phenotypic variations were observed related to geographical origins called as BTV topotypes. The reassortment of BTV genome segments due to both genetic drift and genetic shift resulted in emergence of BTV strains with enhanced virulence^8^ or adaptation into new ecological zones^1^.

Before 2006, transplacental transmission (TPT) of field or wild BTV strains was not reported, unlike modified live or attenuated vaccine viruses of 4 strains namely, BTV-10, -11, -13 and -17 in cattle^1,6^. In August 2006, first TPT of wild BTV-8 was reported in Western Europe^9^. The infection thereafter spread to most parts of the northern Europe and caused disease in sheep and cattle^10,11^. TPT rate varies according to the species, around 10%^10^ to 41.7%^12,13^ in cows, 56 to 69% in ewes^14,15^ and 33% in goats^16^, and stage of gestation^7^. Most important consequences of TPT are birth of BTV positive foetuses and viral persistence in heifers resulted in introduction of BTV in disease free regions^11^.

The drawbacks of experimental studies in natural host species like ruminants includes expensive, ethical issues, inclusion of less number of animals, and difficult in getting BTV sero-negative animals^17,18^. To overcome these constrains, an alternative adult mice model is required. However, adult mice were neither susceptible to BTV infection nor viremia was observed after intravenous (I/V) or subcutaneous (S/C) inoculation due to abnormally excessive levels of type I interferon (IFN) production after BTV infection^17–19^. Calvo-Pinilla *et al*.^17^ characterized an IFN alpha/beta receptors deficient [IFNAR^(−/−)^] adult mice model for *in vivo* study of various BTV serotypes.

BTV serotype-1 was isolated first time from aborted and stillborn goat foetuses from Sardarkrushinagar, Gujarat state, India in 2007^20^. Before 2007, no cases of transplacental infection of BTV serotypes in ruminants have been reported from India. BTV-1 was isolated from foetuses, which indicated the first evidence of TPT of wild-type BTV-1 from India and attenuated or laboratory adapted BTV-1 strains have never been used in this region. This Indian BTV-1 showed unusual clinical manifestation, more than 50% of pregnant goats were aborted or gave birth to dead kids. But to prove this natural case of TPT of Indian BTV-1, experimental studies are completely lacking.

Even though BTV infection has occurred in India since 1964^21^, not much is known about the possible birth defects associated with TPT of Indian BTV in animals, and distribution of viral antigen in reproductive organs has not been described. The clinical, gross, and histopathological findings in pregnant animals infected with wild-type BTV-1 have only been rarely addressed in the literature. To best of our knowledge, there is no published report available regarding localization of BTV-1 antigen in urerus, placenta, ovary and foetuses by immunohistochemistry, humoral and cell mediated immune response, and apoptosis in pregnant animals infected with wild type BTV-1. Researchers are looking to explore the mechanism of transplacental transmission of BTV for better understanding of epidemiology and overwintering mechanism of the virus.

Therefore, the objectives of the present study were to explore the TPT potential of wild Indian BTV-1 at early and mid stages of gestation after experimental infection in IFNAR1-blocked mice. The present study, first time describes the pathological consequences associated with TPT of BTV-1 infection. This study also demonstrated the distribution of BTV-1 antigen in reproductive organs, immune cell kinetics and apoptosis in BTV-1 infected pregnant animals during early and mid stages of gestation.

## Materials and Methods

### Animals

The female virgin Swiss albino mice of 6–8 weeks old were procured from Laboratory Animal Resource (LAR) Section, ICAR-Indian Veterinary Research Institute (ICAR-IVRI), Izatnagar. The animals were kept in polypropylene cages at room temperature (RT; 24 ± 10 °C) and relative humidity of 60 ± 10% with 12/12 h light/dark cycle, and provided feed and water ad libitum. The mice were maintained in insect proof accommodation of Experimental Animal Facility of Centre for Animal Disease Research and Diagnosis (CADRAD), ICAR-IVRI, Izatnagar. All the experiments were performed in accordance to the regulations and guidelines approved by the Institute Animal Ethics Committee (IAEC), ICAR-IVRI, Izatnagar [Approval No. F26-1/2015-16/JD(R)]. All animal procedures were conducted in accordance with the Committee for the Purpose of Control and Supervision on Experiments on Animals (CPCSEA) guidelines (2003).

The stage of estrous cycle was identified by visual examination of vagina, based on the criteria described by Champlin *et al*.^22^. The mice in proestrus and estrus stages were kept for breeding. Two females and one adult fertile male mice of the same strain were kept for mating (2:1 ratio). During the mating period, vagina was examined every morning for the presence of copulatory plug and the presence of sperm was confirmed microscopically using vaginal swabs. The day of finding of copulatory plug was considered as 1 gestation day (GD).

## Experimental Design

### Experiment-1

After confirmation of copulatory plug, mice were inoculated intravenously (I/V) with 50 μl of inoculum containing 1 × 10^6^ TCID_50_/ml of BTV-1 at 1 GD and acted as infected group. The mice were injected with mouse anti-mouse interferon α/β receptor (IFNAR1 subunit) monoclonal antibody (Prod. No. I-401; Clone: MAR1-5A3; Leinco Technologies, Inc., St. Louis, Missouri, USA) at a dose rate of 2.5 mg/mouse intraperitoneally (I/P) at 24 hours before BTV-1 infection^23,24^. The control mice were inoculated with 50 μl of uninfected tissue culture medium, I/V and anti-mouse IFNAR1 monoclonal antibody (2.5 mg/mouse, I/P) before 24 h of PBS and acted as placebo. The control mice were housed in a separate pen without contact with BTV infected animals. Three mice from BTV-1 inoculated and two mice from control pregnant groups were sequentially euthanized by cervical dislocation on 1 (2 GD), 2 (3 GD), 3 (4 GD), 5 (6 GD), 7 (8 GD), 9 (10 GD), 12 (13 GD), 15 (16 GD), 18 (19 GD), and 19/20 (20/21 GD) days post infection (dpi). The dams and all foetuses were examined thoroughly during post-mortem and body weight of foetuses was recorded.

### Experiment-2

The mid stage pregnant mice were inoculated I/V with 50 μl of inoculum containing 1 × 10^6^ TCID_50_/ml of BTV-1 at 8 GD and acted as infected group. The mice were injected with anti-mouse IFNAR1 monoclonal antibody at a dose rate of 2.5 mg/mouse, I/P at 24 hours before BTV-1 infection. The control mid pregnant mice received I/V 50 μl of uninfected tissue culture medium and 24 h before, anti-mouse IFNAR1 monoclonal antibody at a dose rate of 2.5 mg/mouse, I/P and acted as placebo. Three mice from BTV-1 inoculated and two mice from control pregnant groups were sequentially euthanized by cervical dislocation on 1 (9 GD), 2 (10 GD), 3 (11 GD), 5 (13 GD), 7 (15 GD), 9 (17 GD), and 12/13 (20/21) dpi. The dams and all foetuses were examined thoroughly during post-mortem and body weight of foetuses was recorded.

### Inocula

The bluetongue virus serotype-1 used in this experiment was isolated from stillborn and aborted fetal spleens of goats in July 2007 at Sardarkrushinagar, Gujarat, India^20^. The BTV-1 had caused 52% of abortions and birth of dead stillborn kids in 3 months of pregnant goats (13/25). The BTV-1 (SKN-10/India/2007) was supplied by Virus Repository of ICAR-All India Network Program on Bluetongue (AINP-BT), ICAR-IVRI, Mukteswar Campus, Uttarakhand, India. BTV-1 was isolated on baby hamster kidney cells (BHK-21) and passaged once on *Culicoides sonorensis* (KC) cell line, to obtain virus stock for inoculation. The virus stock was titrated in BHK-21 cells to determine a titre of TCID_50_/ml by endpoint titration assay and diluted in cell culture medium before inoculation. Serotype specific PCR was performed using BTV-1 segment 2 (VP2) primers to confirm the BTV serotype (Table 1).

**Table 1:** Primers used in this study for amplification of BTV genome.

Gene	Primer sequence	Primer length	Product length (bp)	Annealing temperature (°C)
BTV-1/Seg-2	FP-5′-GGACATCACTTACGAGCAAGG-3′	21	628	64
	RP-5′-CAGTACTCTGAATCACGTGC-3′	20		
NS3/Seg-10*	FP-5′-TGGAYAAAGCRATGTCAAA-3′	19	100	56
	RP-5′-ACRTCATCACGAAACGCTTC-3′	20		
	TaqMan probe^#^ 5′-(6-FAM)-ARG CTG CAT TCG CAT CGT ACG C-(Tamra-Q)-3′			

*R = A + G, Y = C + T.

^#^The oligo sequence has been labeled with reporter FAM (Carboxyfluorescein) at 5′ end and quencher Tamra at 3′ end. The sequence is a part of segment 10 (NS3), the conserved region present in all BTV serotypes.

### Clinical observations

The pregnant mice were observed daily and recorded for mortality and clinical signs like dullness, anorexia, ocular and nasal discharges, respiratory signs, abortion, stillbirth, staining around the perineum, behaviour and nervous signs.

### Post-mortem lesions

Systematic post-mortem examination was carried out and gross findings were recorded. The uterus of each dam was examined to record the number of implantation sites, total number of embryos and/or foetuses, number of live and dead embryos and/or foetuses, and number of mummified and/or macerated foetuses. Further, macroscopic abnormalities in the foetuses were checked carefully. The axillary and inguinal lymph nodes, lungs, spleen, heart, thymus, brain, liver, etc. examined for gross lesions. The effect of BTV-1 on early and mid stages of gestation was compared with control uninfected pregnant mice.

### Sample collection

To avoid cross-contamination, control mice were autopsied first and separate sterilized equipments were used for each infected animal for sampling. At euthanasia, blood was collected from hearts of both infected and control mice in EDTA coated vacutainers for haematological studies, fluorescent activated cell sorting (FACS) analysis, and BTV RNA quantification. Blood was also collected in BD Vacutainer^®^ plus plastic SST^™^ with Polymer Gel tubes for serum separation for BTV-1 antibody detection. Tissues from axillary and inguinal lymph nodes, lungs, spleen, heart, thymus, brain, liver, kidneys, uterus, placenta, ovaries, embryos, entire foetuses, and foetal organs were collected in 10% neutral buffered formalin (NBF) for histopathology and immunohistochemisry (IHC), and in RNAlater^®^ (Ambion, USA) for BTV-1 detection, and stored at −80 °C. Care was taken to avoid contamination of foetuses with maternal blood. The spleens were collected in RPMI-1640 medium (Sigma-Aldrich, St. Louis, Missouri, USA) in ice for FACS analysis.

The embryos were too small and difficult to dissect them for individual organ sampling. Therefore, few embryos were fixed as such without dissection in 10% NBF for histopathology and IHC, and few embryos were frozen at −80 °C for virus detection. The contents of each entire embryo was homogenized and treated as one sample. On 20/21 GD only, individual organs of foetuses were sampled.

### Histopathology

The formalin-fixed tissues were cut into pieces of 2 to 3 mm thickness and washed with water, dehydrated in ascending grades of alcohol, and cleared in xylene. The dehydrated tissues were embedded in paraffin and sections of 4–5 μ thickness were cut and stained with haematoxylin and eosin (H&E) as per standard procedure.

### RNA extraction

Total RNA was extracted from EDTA stabilized blood and various tissues preserved in RNAlater^®^ (Ambion, USA) using TRIzol™ reagent (Invitrogen™, ThermoFisher Scientific, Carlsbad, CA, USA) as per manufacturer’s recommendations. The total RNA suspension was treated with RQ1 (RNA Qualified) RNase-Free DNase enzyme (Promega, WI, USA) followed by enzyme inactivation at 65 °C for 10 min to remove the possible traces of genomic DNA. Purity of the RNA was analysed in spectrophotometry and integrity of the RNA was tested by electrophoresis. The final RNA pellet was stored at −80 °C.

### Quantification of BTV genome by real-time qRT-PCR

The viral loads in blood and various tissue samples at specified time points were quantified by Taqman probe based real-time polymerase chain reaction (PCR) using NS3 probe and primers specific for BTV segment 10 (Table 1) as described previously^25^. The QIAGEN^®^ OneStep RT-PCR Kit (Qiagen, Hilden, Germany) was used in CFX96™ Real-Time System (BIO-RAD, USA). A volume of 25 μl of PCR reaction mix was prepared with 2 μg of RNA as template combined with 10 pmol each of forward and reverse primers and probe with other components in the reactions according to the manufacturer’s instructions. The cycling conditions were reverse transcription at 50 °C for 30 min, initial PCR activation at 95 °C for 15 min, template denaturation at 94 °C, primer annealing at 56 °C, extension at 72 °C for 30 sec, and final extension at 72 °C for 10 min. Individual cycle threshold (Ct) values were determined from the point at which the level of fluorescence passed a threshold fluorescence line. The real-time qRT-PCR amplification was confirmed by dissociation curve at the end of reaction. The real-time PCR products were further confirmed by 1.5% agarose gel electrophoresis and visualized under UV light after staining with ethidium bromide. The standard curve was used for the quantification of BTV RNA in samples.

### Immunohistochemistry

Duplicate formalin fixed tissue sections were taken on (3-Aminopropyl)triethoxysilane (APES, Sigma Aldrich, St. Louis, Missouri, USA) coated glass slides. BTV-1 antigen localisation was demonstrated in lymph nodes, lungs, spleen, brain, placenta, uterus, ovary, and foetal tissues of pregnant mice by indirect immunoperoxidase technique (IPT) using anti-BTV antibodies. The sections were deparaffinised and rehydrated in graded alcohol followed by gently rinsing in distilled water (DW). The antigen retrieval was performed by microwave in 10 mM tri-sodium citrate buffer (pH 6.0) for 15 min (3 cycles of 5 min each) to unmask the antigenic sites, and sections were left to cool at RT for 20 min. Then the slides were washed with PBS (pH 7.4; 3 times of 5 min each). Endogenous peroxidase activity was blocked by incubating with freshly prepared 3% hydrogen peroxide in 80% methanol for 30 min in dark chamber and sections were washed in PBS (3 times of 5 min each). Further, the sections were incubated with 5% normal goat serum (Invitrogen; product no. 016201) in PBS for 1 hour at RT in humidified chamber for blocking of non-specific antigen binding sites, followed by washing in PBS (3 times of 5 min each). Primary antibody against BTV-1 core antigen raised in rabbit at 1:20 dilution in 1% bovine serum albumin was incubated on the sections overnight in humidified chamber at 4 °C. Then, the slides were washed thrice with PBS and incubated with peroxidase-conjugated goat anti-rabbit secondary antibody (Sigma-Aldrich, St. Louis, Missouri, USA) for 1 hour at 37 °C, followed by washing thrice in PBS for 5 min each. To demonstrate the immunolabelling, the slides were incubated with ImmPACT^™^ DAB peroxidase substrate (Cat No. SK-4105; Vector Laboratories, Inc., Burlingame, CA, USA) for 30–60 seconds and sections were washed with de-ionised water. The slides were counter stained with Mayer’s haematoxylin and rinsed in tap water. The sections were covered with CC/Mount^™^ aqueous mounting medium (Sigma-Aldrich, St. Louis, Missouri, USA) and then dried at RT for 45 min to 1 hour. The sections were examined under the microscope for positive signals. Slides prepared from known BTV positive samples were used as positive control.

### BTV specific antibody detection

The appearance of group-specific antibodies against BTV were measured in serum from BTV infected and control pregnant mice using a commercial BTV VP7 cELISA Kit (Cat No. 287-5; VMRD Inc., Pullman, WA, USA) according to the manufacturer’s instructions. The results of cELISA were expressed as percentage inhibition (PI) values, as follows: [1 − (OD_620 nm_ of test serum/OD_620 nm_ of negative control serum)] × 100. The test samples were considered positive when the PI value of the sample is equal to or more than 50%.

### Haematology

At each sacrificed interval, blood samples were analysed for red blood cell (RBC) count, total leukocyte count (TLC), packed cell volume (PCV), haemoglobin (Hb), total platelet count (TPC), differential leucocyte count (DLC), mean corpuscular volume (MCV), mean corpuscular haemoglobin (MCH), and mean corpuscular haemoglobin concentration (MCHC) using automated blood analyzer (Horiba, ABX Micros ESV60, Japan).

### Separation of peripheral blood mononuclear cells (PBMCs)

Anti-coagulated blood was pooled according to the experimental groups for isolation of PBMCs as described previously^26^ and diluted with 1x Dulbecco’s phosphate buffered saline (D-PBS; pH 7.2; ratio 1:1) without calcium and magnesium. The blood was subjected to density gradient centrifugation method using Histopaque^®^ (Sigma Aldrich, St. Louis, Missouri, USA) with a density of 1.083 g/ml. Equal volume of diluted blood (ratio 1:1) was slowly layered over the Histopaque^®^ without mixing and centrifuged at 2000 rpm for 15 min at RT, resulting in separation of PBMCs at plasma-histopaque interface. The PBMC layer was collected carefully without disturbing the plasma and histopaque layer, and washed twice in isotonic PBS at 2000 rpm for 5 min. To remove the traces of RBCs, 1 ml of 1x RBC lysis buffer was added and incubated for 10 min in ice and centrifuged at 2000 rpm for 5 min. The final PBMC pellet was resuspended in 200 μl of stain buffer (BD Pharmingen™, BD Biosciences, San Jose, CA, USA) containing heat-inactivated foetal bovine serum (FBS) and sodium azide to maintain cell viability and maximise fluorescence signal intensity. Cell number and viability were counted by trypan blue dye exclusion method in Neubauer chamber after 1:10 dilution with trypan blue solution (0.01%) and the cell concentration was adjusted to 1 × 10^6^/ml of cells.

### Separation of splenocytes

Splenocytes were separated from spleen by mincing into small pieces as described previously^26^. The splenocytes were squeezed from the splenic capsule in PBS (pH 7.4) through 70 μm nylon mesh cell strainer in a Petri dish to create a single cell suspension by gently mashing spleen pieces with rubber end of a plunger from 1 ml tuberculin syringe. The suspensions of dispersed cells were again filtered through cell strainer into a sterile centrifuge tube on ice. Then, cells were centrifuged at 2000 rpm for 5 min at 4 °C and supernatant was discarded. The cell pellet was re-suspended in chilled 1x RBC lysis buffer to remove traces of RBCs and centrifuged at 2000 rpm for 5 min at 4 °C. Supernatant was discarded and the final cell pellet was resuspended in 200 μl of stain buffer. Cell number and viability were counted by trypan blue dye exclusion method in Neubauer chamber and the cell concentration was adjusted to 1 × 10^6^/ml of cells.

### Fluorescence-activated cell sorting (FACS)

Mouse T lymphocyte subset monoclonal antibody cocktail with isotype control labelled with PE-Cy™7 CD3e, PE CD4, and FITC CD8 (Cat No. 558391) for estimation of CD4^+^ and CD8^+^ T lymphocyte subsets in PBMCs and splenocytes, and PE-Cy™7 mouse antimouse NK-1.1, clone PK136 (Cat no. 552878, BD Pharmingen™, BD Biosciences, San Jose, CA, USA) were used in the study. Flow cytometric analysis was performed for evaluation of percentage of CD4^+^ and CD8^+^ T lymphocyte subsets, and NK cells in PBMCs and splenocytes in the infected and control groups. The protocol for FACS analysis was followed: 15 μl of mouse T lymphocyte subset antibody cocktail was added in one set of sample, and 15 μl of anti-mouse NK-1.1 antibody was added in another set of sample as per the manufacturer’s recommendations. Samples were then mixed gently and incubated at RT in dark for 45 min. The tubes were kept in icebox in dark and taken for FACS analysis. Ten thousand cells were counted and analysis was performed in the BD FACS Canto II Flow Cytometer using FL1 (CD8-FITC), FL2 (CD4-PE) and FL3 (NK-PE-Cy^™^7) band pass filters. The data were analyzed using BD CellQuest™ Pro Software (BD Bioscience, San Jose, CA, USA).

### Analysis of early apoptotic cells in PBMCs

Following BTV-1 infection, apoptosis was studied in PBMCs at the specified intervals using TACS^®^ Annexin V-FITC Apoptosis Detection Kit (Cat. No: 4830-01-K, Trevigen, Inc) and analyzed using flow cytometry. Following protocol was used to process the samples for FACS analysis; the isolated PBMCs (1 × 10^6^/100 μL of cells per sample) were washed with chilled 1x PBS buffer on ice. 100 μL of Annexin V incubation reagent was prepared freshly by mixing 10 μL of 10x binding buffer, 10 μL of propidium iodide (PI), 1 μl of Annexin V-FITC, and 79 μl of deionized DW. The cells were resuspended and incubated with 100 μl of prediluted Annexin V reagent in the dark for 15 min at RT and 400 μl of 1x binding buffer was added to the samples and tubes were kept in icebox in dark and taken for FACS analysis within one hour for maximal signal. Ten thousand cells were counted and analysis was performed in the BD FACS Canto II Flow Cytometer (BD Biosciences, USA) using FL1 (Annexin V-FITC-early apoptotic) and FL3 (PI-late apoptotic or necrotic) band pass filters. The data were analyzed using BD CellQuest™ Pro Software (BD Bioscience, San Jose, CA, USA) and the results were plotted as percentage of annexin-V^+^PI^−^ cells.

### Detection of apoptotic cells in formalin fixed tissues

Duplicate formalin fixed tissue sections were taken on APES coated glass slides for *in situ* detection of apoptotic cells using *In Situ* Cell Death Detection Kit, AP (Sigma-Aldrich, St. Louis, Missouri, USA) as per manufacturer’s protocol. Briefly, formalin fixed paraffin embedded tissues were deparaffinised and rehydrated through descending graded alcohol. The sections were incubated with proteinase-K solution (20 μg/ml) for 20 min at 37 °C. The sections were washed thrice with PBS for 5 min each, incubated with 50 μl of TUNEL reaction mixture containing 5 μl of enzyme solution and 45 μl of label solution, and incubated for 1 hour at 37 °C in humidified chamber in dark. The slides were again washed thrice in PBS and viewed under fluorescence microscope for the positive signals.

### Alizarin red staining

Alizarin red staining was performed to characterize the skeleton deformities in the foetuses born from BTV-1 infected and control pregnant mice at 20/21 GD. The procedure for alizarin red staining for each group was followed as described by Ovchinnikov^27^. The length of the foetuses, different bones in forelimb (scapula, humerus, radius and ulna) and hind limb (femur, tibia and fibula), vertebral column length (cervical–sacral), and skull (length and diameter) were measured using Olympus SZ61 stereo zoom microscope with Olympus SZ2-ILST LED illumination stand.

### Statistical analysis

The data were analyzed using GraphPad Prism, Version 5.0 (GraphPad Software Inc, San Diego, CA, USA). The mean of the infected and control groups at specified time intervals were calculated and expressed as mean ± standard error of the mean (SEM). Unpaired t test, one-way ANOVA with Tukey’s post-test, two-way ANOVA with Bonferroni post-test were used at suitable places to know the effects of BTV-1 in early and mid stages of gestation and uninfected control pregnant groups. For all comparisons, differences were considered significant at P < 0.05.

## Results

### Clinical signs

The clinical signs were recorded over a period of 20/21 GD in both infected and control groups. The BTV-1 infected IFNAR1-blocked early stage pregnant mice showed signs of dullness and depression on 2 dpi. The clinical signs became more prominent in dams on 3 dpi onwards with conjunctivitis, anorexia, and weight loss. Signs of abortions or stillbirth like fetal discharge, bleeding, and bloody staining around the vagina were noticed from 7 dpi onwards. The clinical signs were more severe on 7 to 15 dpi due to disease progression but after 15 dpi, clinical signs gradually subsided.

The prominent clinical signs in dam infected with BTV-1 during mid stage of gestation (8 GD) were loss of appetite (2 dpi), depression (3 dpi), conjunctivitis (5 dpi), ocular and nasal discharges (5 dpi), weight loss, and sluggish movements with huddling tendency (7 dpi). Signs of abortions or stillbirth were noticed from 5 dpi onwards. The foetuses born at 20/21 GD showed neurological signs. Mortality in dams was not observed during the experimental period in both groups of pregnant mice. The control groups similarly did not show any clinical signs or mortality during the experimental period.

Body weights of foetuses in the various groups sacrificed on 20/21 GD were depicted in Supplementary Fig. S1. The foetuses born from BTV-1 infected groups showed significant decrease in body weights compared to uninfected control foetuses. Foetuses from mid gestation showed non-significantly decreased body weight compared to early gestation.

### Gross lesions in early gestation

**Figure 1:**
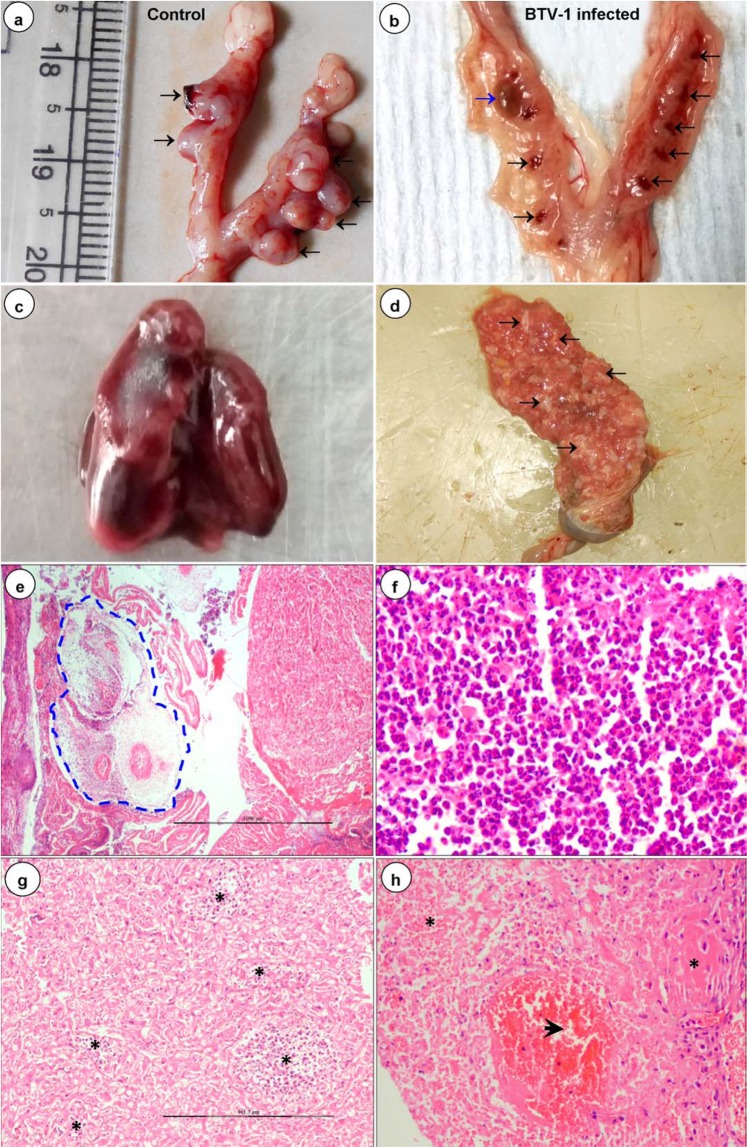
Gross (**a–d**) and histopathological (**e–h**) lesions in BTV-1 infected early gestation group. **(a)** Normal embryos (arrow) in the uterine lumen of uninfected control mice on 9 dpi. **(b)** Loss of embryos leaving only implantation sites (black arrow) in BTV-1 infected mice on 9 dpi. Oedematous and necrosed uterine mucosa with dead, shrunken, and discoloured embryo (blue arrow). **(c)** Severe congestion, haemorrhages and mummification of foetus in BTV-1 infected mice on 15 dpi. **(d)** Stomach of BTV-1 infected dam had digested contents of aborted foetuses (arrow) on 15 dpi. **(e)** Advanced stage of resorpting embryo (blue dotted line) infiltrated with inflammatory cells and necrotic placenta on 12 dpi. H&E x40. **(f)** Higher magnification of necrotic placenta showed severe infiltration of neutrophils and few macrophages. H&E x400. **(g)** Multifocal necrotic and inflammatory cell infiltrated areas (asterisk) in labyrinth zone of placenta on 15 dpi. H&E x100. **(h)** Severe haemorrhages (arrow) and necrosis (asterisk) in the mucosa and submucosa of uterus on 9 dpi. H&E x200.

In dams infected at early stage of gestation showed reduced implantation sites, early embryonic deaths, abortions, haemorrhages, and necrosis in the placentae and uteri. In uterine lumen, deciduas had necrosis with central depression. Early embryonic deaths characterized by loss of embryos (abortions), discoloured and shrunken embryos, and detachment from implantation sites resulted in without embryos on 9 dpi (Fig. 1b). The uterine mucosa was found edematous and often necrosed with engorged uterine arteries (Fig. 1b). Discolouration of amniotic fluid, development of gelatinous consistency, detachment and/or absence of placentomes was noticed on 12 dpi onwards. Early embryonic death and fetal resorption without uterine swelling was found in one uterine horn with uneven uterine swelling in another horn was noticed in dams (Supplementary Tables S1, 2). Severe congestion, haemorrhages, necrosis, and mummification of foetuses were noticed from 15 dpi onwards (Fig. 1c).

**Figure 2:**
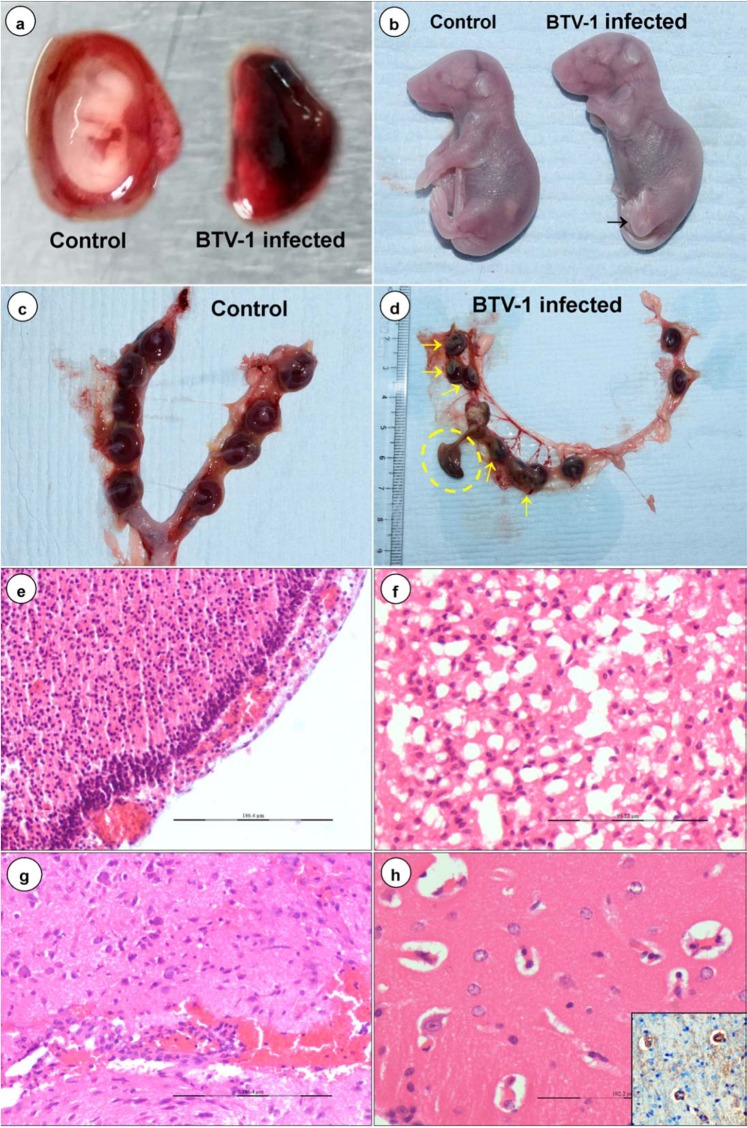
Gross (**a–d**) and histopathological (**e–h**) lesions in BTV-1 infected mid gestation group. **(a)** Hemorrhagic, dead and resorption of embryo in BTV-1 infected group on 7 dpi. **(b)** BTV-1 infected foetus showed micromelia and syndactyly (arrow) on 13 dpi. **(c)** Symmetrical distribution of reddish discoid shape placenta of uninfected pregnant mice on 13 dpi. **(d)** Shrunken discoloured and necrosis of placenta (yellow arrow) with fetal mummification (yellow circle) in BTV-1 infected group on 13 dpi. **(e)** Marked thickening of meninges due to congestion, haemorrhages, edema, and infiltration of inflammatory cells on 9 dpi. H&E x200. **(f)** Numerous empty, irregular shaped vacuoles indicated severe diffuse cerebral edema on 12 dpi. H&E x400. **(g)** Necrosis of endothelial cells, haemorrhages and mild perivascular cuffing in brain on 12 dpi. H&E x200. **(h)** Perivascular edema and swollen endothelial cells occluding the lumen of capillaries on 13 dpi. H&E x400. Inset showing localization of BTV in endothelial cells. IP-DAB-MH x200.

It was noticed that in the stomach of BTV-1 infected dams contained remnants of aborted embryos and/or foetuses (Fig. 1d). Also there were enlarged and congested spleens and axillary and inguinal lymph nodes. Congestion and petechial haemorrhages in different organs like lungs, myocardium, kidneys, and liver were also observed. The control animals did not reveal any significant changes (Fig. 1a).

### Gross lesions in mid gestation

The dams infected at mid stage of gestation revealed severe congestion, haemorrhages, and edema of brain in foetuses. Severe congestion of uterine horns was noticed on 5 dpi. Haemorrhages and resorption of embryos were noticed on 7 dpi (Fig. 2a). Blood clot formation was observed in amniotic cavity on 9 dpi. Discolouration of uterus with unequal and asymmetrical embryonic distribution in the uterine horns was observed on 13 dpi. Placentas wall become shrunken, discoloured, congested, haemorrhagic and necrotic with adherence of yolk sacs to the chorionic surface along with fetal mummification on 13 dpi (Fig. 2d). Developmental anomaly like micromelia with syndactyly characterized by shorter hind limb with fusion of toes was observed in one foetus on 13 dpi (Fig. 2b).

There were enlarged and edematous axillary and inguinal lymph nodes, and focal areas of consolidation in lungs on 7 dpi in dams. Markedly enlarged and congested spleen was observed on 9 dpi. Congestion was also observed in tracheas, hearts, livers, and kidneys on 5 dpi onwards. The reproductive organs and foetuses of uninfected control mice did not show any significant gross lesions (Fig. 2b,c).

### Histopathological lesions in early gestation

**Figure 3:**
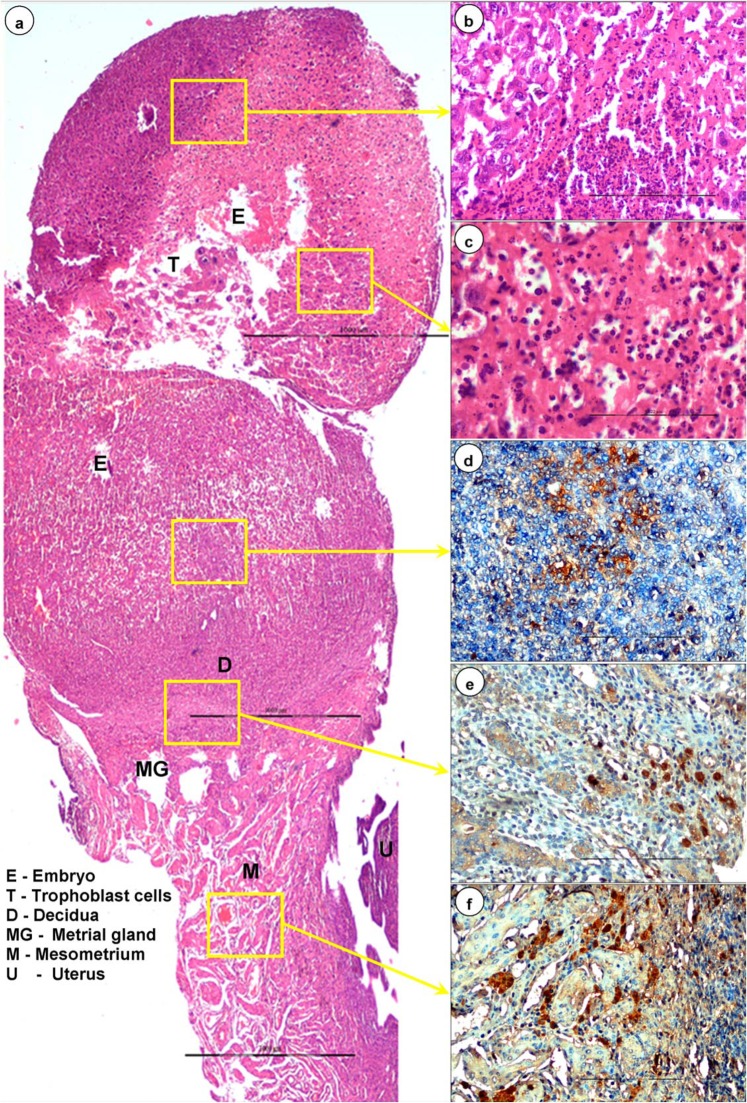
Histopathological (**a–c**) and immunohistochemical (**d–f**) findings in BTV-1 infected early gestation group. **(a)** Dead and resorpting embryo showed massive necrotic areas with infiltration of inflammatory cells on 7 dpi. **(b,c)** Severe necrosis of embryonic stromal cells with basophilic nuclear remnants and infiltration of neutrophils and lymphocytes in the embryo on 7 dpi. **(d)** Positive immunolabelling of BTV-1 antigen in endometrial stromal cells and decidual cells. IP-DAB-MH x200. **(e)** Positive immunolabelling of BTV-1 antigen in metrial glands, decidual and embryonic stromal cells. IP-DAB-MH x200. **(f)** Positive immunolabelling of BTV-1 antigen in cells of mesometrium and metrial glands. IP-DAB-MH x200.

The tissues from dead and resorpting embryos revealed massive necrotic foci with basophilic nuclear remnants and marked infiltration of neutrophils and lymphocytes on 7 (Fig. 3a-c) and 9 dpi. The embryos retrieved later were markedly infiltrated with macrophages, plasma cells and neutrophils on 12–18 dpi (Fig. 1e,f). Marked congestion in the lamina propria of yolk epithelium was noticed on 12 dpi onwards and necrotic cellular debris consisting of predominantly neutrophils with a few macrophages in the placenta and also in its labyrinth zone were observed on 15 dpi (Fig. 1g). Severe congestion, haemorrhages, and necrosis in mucosa and submucosa of uteri were noticed on 7 dpi onwards (Fig. 1h). Vacuolar degeneration and dilatation of endometrial glands in lamina propria of uteri were also observed on 12–15 dpi. Apoptosis and degeneration of tertiary follicles of ovary were observed on 19/20 dpi.

Lymphoid depletion in spleens, lymph nodes and thymus of BTV-1 infected dam on 7 dpi and foetuses on 19/20 dpi were noticed. White pulp hyperplasia in spleens of dams was noticed on 15 dpi. Engorgement of inter-alveolar capillaries and haemorrhages in lungs of dams and foetuses were observed. Interstitial pneumonia and peribronchiolar lymphocytic infiltrations were observed in dams on 7–9 dpi. Multifocal interstitial lymphocytic infiltration was noticed in myocardium of dams on 7–9 dpi. The uninfected control animals did not reveal any histopathological lesions.

### Histopathological lesions in mid gestation

**Figure 4:**
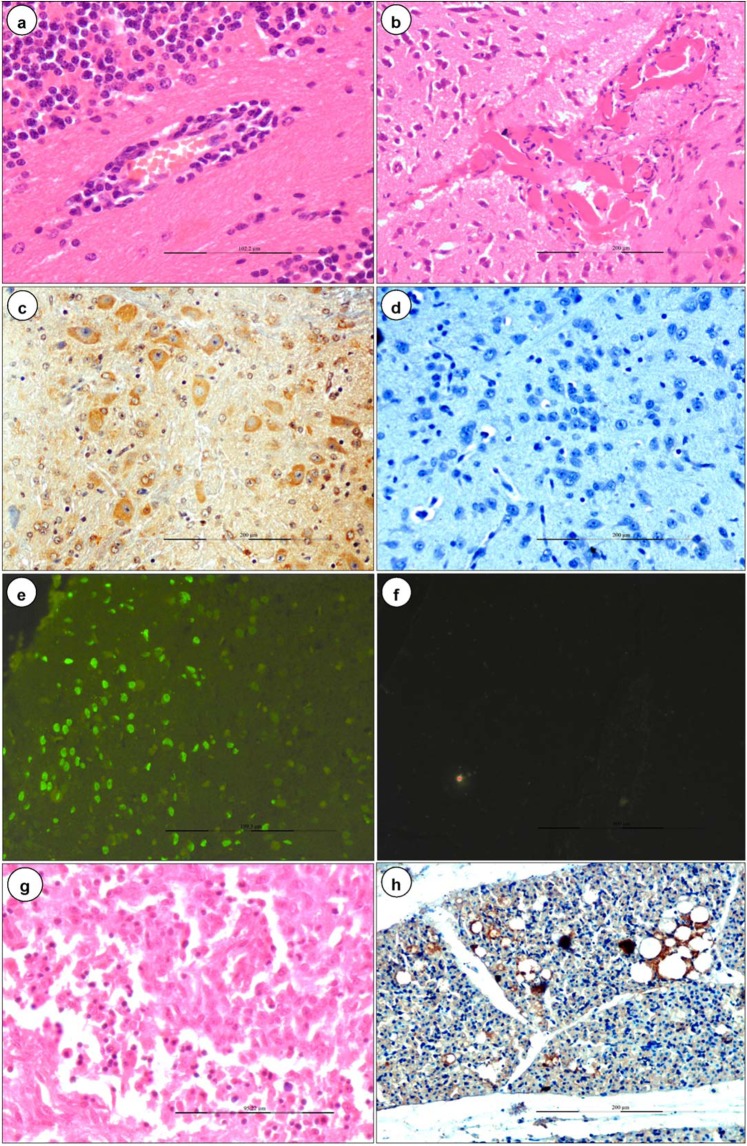
BTV-1 induced lesions (**a,b,g**), immunohistochemical localization (**c,d,h**) and apoptosis (**e,f**) in the foetuses born from dams infected during mid stage of gestation. **(a)** Perivascular cuffing with lymphocytes, congestion and swollen endothelial cells on 18 dpi. H&E x400. **(b)** Necrotizing encephalitis in cerebrum: Eosinophilic areas of malacia (necrosis) surrounded by glial cells on 20 dpi. H&E x200. **(c)** Positive immunolabelling of BTV-1 antigen in the cytoplasm of neurons, endothelial cells, and glial cells in brain. IP-DAB-MH x200. **(d)** Brain did not show positive immunoreaction in uninfected control mice. IP-DAB-MH x200. **(e)** Apoptotic cells showed green fluorescence signals in the brain of BTV-1 infected group. FITC x200. **(f)** Negative control: Brain showing no positive immunoreaction for BTV-1 antigen. **(g)** Infiltration of plasma cells, macrophages and lymphocytes in pleural space of lungs on 18 dpi. H&E x200. **(h)** Positive immunolabelling of BTV-1 antigen in alveolar epithelial cells of lung on 18 dpi. IP-DAB-MH x 200.

Meningo-encephalitis, vascular lesions (congestion, haemorrhages, and endothelial cell swelling), and vacuolations in brain parenchyma were the most prominent microscopic findings in BTV-1 infected foetuses (Fig. 2e-h). Significantly reduced mass of brain parenchyma was observed in BTV-1 infected group on 12/13 dpi. Marked thickening of meninges due to congestion of blood vessels, infiltration of inflammatory cells and swollen endothelial cells occurred from 9 dpi onwards (Fig. 2e). Marked cerebral edema characterized by numerous empty, irregular shaped vacuoles in the grey and white matter of cerebrum, cerebellum and hippocampus was observed on 12/13 dpi (Fig. 2f). Perivascular edema and haemorrhages, necrosed and swollen endothelial cells occluding the lumina of capillaries were also observed on 13 dpi (Fig. 2g,h). Perivascular cuffing with lymphocytes and necrotizing encephalitis in cerebrum with homogenous eosinophilic areas of malacia (necrosis) surrounded by glial cells were observed on 12/13 dpi (Fig. 4a,b). Diffuse and focal infiltration of glial cells was noticed in cerebrum. Marked dilatation of lateral ventricles of brain with haemorrhages, and severe congestion in subventricular zone were recorded. In fetal lungs, infiltration of plasma cells, macrophages, and lymphocytes in pleural spaces was noticed (Fig. 4g).

Marked congestion and irregular dilatation of spiral arteries, and haemorrhages with disruption of decidua basalis, basal zone and labyrinth zone of placenta were observed on 9 dpi (Supplementary Fig. S2a). Metrial gland area of the placenta had necrosis and was heavily infiltrated with neutrophils, macrophages, and few plasma cells and lymphocytes on 9 dpi (Supplementary Fig. S2a,b). Necrosis of labyrinth zone and trophoblast cells, and decreased thickness of trophoblastic septa with absence of blood (RBCs) in maternal and fetal sinusoids in labyrinth zone of placenta were observed on 7 dpi onwards. In yolk sac epithelium, vacuolar degeneration, congestion, fusion and necrosis, and leukocytic infiltration in lamina propria were observed on 9 dpi onwards (Supplementary Fig. S2c). In uteri, congestion of spiral arteries with marked infiltration of lymphocytes in perivascular areas, mesometrium and myometrium were observed on 7 and 9 dpi (Supplementary Fig. S2d). Apoptotic and degenerating tertiary follicles of ovary were observed on 7-12/13 dpi.

Lymphoid depletion in cortex of lymph node and white pulp of spleen was observed in the BTV-1 infected dam on 3 dpi. Hyperplasia of white pulp of spleen was observed on 5 dpi onwards. Congestion and haemorrhages in cortical and medullary areas, degeneration and necrosis of proximal and distal convoluted tubules were recorded in kidneys on 7–9 dpi. Mononuclear cell infiltrations were noticed in myocardium on 5 to 7 dpi. In liver, lymphocytic infiltrations around portal areas, hepatocyte degeneration and centrilobular micro and macro vacuolations in the hepatocytes were observed on 7 to 9 dpi.

### Quantification of BTV genome by qRT-PCR

**Figure 5:**
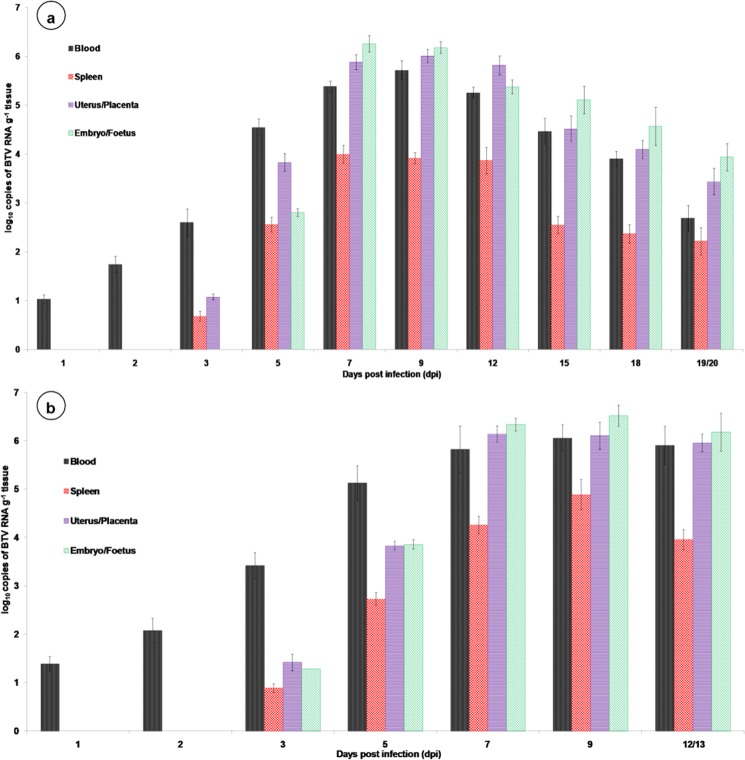
Quantification of BTV RNA in blood, spleen, uterus/placenta and embryo/foetus of pregnant mice infected with BTV-1 during early (**a**) and mid (**b**) stages of gestation at various time points using TaqMan probe-based real-time PCR assay. Results are presented as bar diagram with mean value and standard error of the mean (SEM) at each time point (n = 3). Virus load in blood is given as log_10_ copies of viral RNA ml^−1^ of samples and virus load in tissues is given as log_10_ copies of viral RNA g^−1^ tissue.

The BTV was detected in 12 of 21 (57.14%) samples from uterus/placenta of dams infected during early stage of gestation. Infection at mid stage of gestation resulted in transmission of BTV in 15 of 21 (71.43%) samples from placenta of dams. The BTV viral RNA quantified in reproductive organs of dams like uterus/placenta, embryo/foetus, and ovary of early and mid gestation group have been summarized in Fig. 5a,b. Similarly, the BTV viral RNA quantified from blood, spleen, lungs, thymus, heart, and brain of dams from early and mid stages of pregnant group at specified time points (Tables 2, 3; Fig. 5a,b). The BTV viral RNA quantified in fetal organs like brain, thymus, lungs, heart, and spleen born from early and mid stages of gestation (Supplementary Fig. S3). The BTV RNA was detected in blood on 1 dpi and highest copy number was detected on 9 dpi. In uterus and placenta, BTV RNA was detected from 3 dpi onwards and highest copy number was detected on 9 dpi (early) and 7 dpi (mid gestation). In embryo/foetus, BTV RNA was detected on 5 dpi (early), and 3 dpi (mid gestation). In ovary, lungs, thymus, and heart, BTV was detected from 3 dpi onwards in both groups except brain on 5 dpi in early gestation. The uninfected control mice were negative for BTV RNA by qRT-PCR.

**Table 2:** Quantification of BTV RNA from various tissues of pregnant mice infected with BTV-1 during early stage of gestation at various time points using TaqMan probe-based real-time PCR assay.

Tissue names	Days post infection									
	1	2	3	5	7	9	12	15	18	19/20
Lungs	ND	ND	0.72	1.15	2.56	3.69	2.96	2.24	1.59	1.26
Thymus	ND	ND	0.96	2.36	3.94	3.85	3.23	2.85	1.86	1.63
Heart	ND	ND	1.93	3.29	4.27	4.88	3.94	3.07	2.58	2.16
Brain	ND	ND	ND	0.68	0.92	1.84	1.34	1.19	1.16	1.09
Ovary	ND	ND	0.87	2.79	4.53	4.19	3.52	3.26	2.76	2.35

n = 3 at each time point; ND: Not detected; virus load in tissues is given as log10 copies of viral RNA g^−1^ tissue.

**Table 3:** Quantification of BTV RNA from various tissues of pregnant mice infected with BTV-1 during mid stage of gestation at various time points using TaqMan probe-based real-time PCR assay.

Tissue names	Days post infection						
	1	2	3	5	7	9	12/13
Lungs	ND	ND	0.87	1.36	2.78	3.82	3.06
Thymus	ND	ND	1.03	2.53	3.68	4.16	3.17
Heart	ND	ND	2.16	3.7	4.72	4.9	3.67
Brain	ND	ND	0.73	1.18	1.85	2.1	2.35
Ovary	ND	ND	1.17	2.86	4.6	3.93	3.72

n = 3 at each time point; ND: Not detected; virus load in tissues is given as log10 copies of viral RNA g^−1^ tissue.

### Immunohistochemistry

In early stage of pregnant mice, positive immunolabelling of BTV-1 antigen was noticed in embryonic stromal cells, decidual cells (Fig. 3d), cells of mesometrium and metrial glands (Fig. 3e,f), and infiltrated lymphocytes in the necrosed embryos on 5 dpi onwards. Foetuses from mid stage of pregnant mice showed BTV-1 antigen in cells of dura mater and pia mater of brain. BTV-1 antigen was also demonstrated in swollen endothelial cells of capillaries, cytoplasm of neurons, and glial cells in the brain (Fig. 4c). BTV-1 antigen was also demonstrated in alveolar epithelial cells of fetal lungs of both groups on 18 dpi (Fig. 4h).

**Figure 6:**
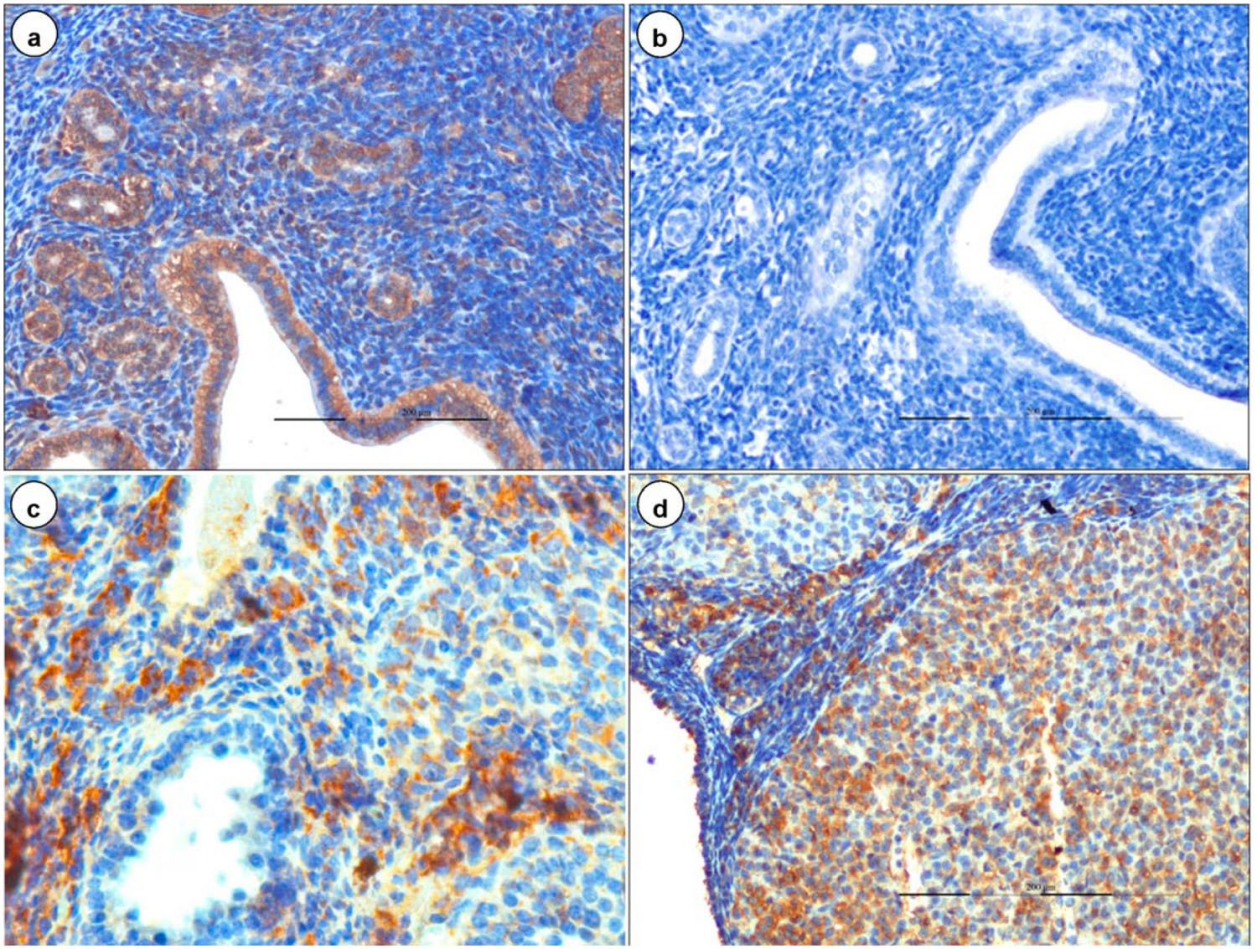
Immunohistochemical localization of BTV-1 antigen in early gestation group. **(a)** Positive immunolabelling of BTV-1 antigen in luminal epithelium, endometrial glands, and stromal cells of uterus on 7 dpi. IP-DAB-MH x200. **(b)** Uterus did not show positive immunoreaction in uninfected control mice. IP-DAB-MH x200. **(c)** Strong positive immunolabelling of BTV-1 antigen in granulosa cells, theca interna and externa of follicles, and cortical stromal cells of ovary. IP-DAB-MH x200. **(d)** Strong positive immunolabelling of BTV-1 antigen in cortical stromal cells, small theca lutein cells, and large granulosa lutein cells of corpus luteum of ovary. IP-DAB-MH x200.

Both early and mid stages of pregnant mice showed BTV-1 antigen in luminal and glandular epithelium and stromal cells in the lamina propria of the uterus (Fig. 6a). Strong positive immunolabelling of BTV-1 antigen was demonstrated in granulosa cells, theca interna and externa of secondary and tertiary follicles, cortical stroma, and small theca lutein cells and large granulosa lutein cells of corpus luteum (CL) of ovary (Fig. 6c,d). BTV-1 antigen was demonstrated in yolk sac epithelial cells of placenta. In reproductive organs (uterus, placenta, ovary, and embryo), the intensity of immunohistochemical signals were more in group infected during early gestation than during mid gestation. In fetal brain, intensity of immunohistochemical signals were more in mid gestation. Uterus, brain, and yolk sac epithelial cells of placenta of uninfected control mice did not show any positive immunoreactions (Fig. 6b).

### Humoral immune response

Humoral immune response was assessed by detecting BTV specific antibodies. The pregnant animals infected with BTV-1 during early stage of gestation were negative for BTV specific antibodies up to 5 dpi. The BTV infected dam serum samples showed positivity with PI values of more than 50% on 7 dpi and levels reached peak on 15 dpi (83.24%), and thereafter started to decline. During infection at mid stage of gestation, serum samples were found negative for BTV antibodies up to 3 dpi, and 5 dpi onwards showed positive. The antibody levels were significantly increased on 9 dpi (84.26%), and thereafter started to fall. The uninfected control animals of both groups were remained negative for BTV antibodies with less than 50% PI values.

## Assessment of Cell Mediated Immunity

### Haematology

BTV-1 infected mice during early stage of gestation showed constantly decreased TLC (P < 0.001) and lymphocyte counts (P < 0.05) up to 7 dpi. Subsequently, TLC and lymphocyte counts started to increase to non-significantly levels on 15 dpi (Supplementary Fig. S4a,c). The neutrophil counts in BTV-1 infected group were non-significantly increased on 5–9 dpi (Supplementary Fig. S4e). Monocyte counts in early stage infected group was decreased on 7 dpi and thereafter increased. The eosinophil and basophil counts did not show any significant variations. The RBC, TPC, Hb, and PCV values showed decreasing trend up to 7 dpi and thereafter increased to normal on 19/20 dpi. The MCV and MCHC values remained almost normal in both groups.

BTV-1 infected mice during mid stage of gestation showed significantly decreased TLC (P < 0.05) and non-significantly decreased lymphocyte counts on 7 dpi. The TLC values, however, became normal with significantly (P < 0.05) increased lymphocyte counts on 12/13 dpi (Supplementary Fig. S4b,d). Neutrophil counts showed non-significant changes (Supplementary Fig. S4f). Monocyte counts in infected group increased non-significantly on 7–9 dpi. Eosinophil and basophil counts did not show any significant changes in the infected mice. The RBC, TPC, Hb, and PCV values revealed slight decrease on 7–9 dpi in BTV-1 infected group. The MCV and MCHC values did not show any significant change in BTV-1 infected mice than control group throughout the experiment.

### Kinetics of CD4^+^ and CD8^+^ T cells in spleen and PBMCs

**Figure 7:**
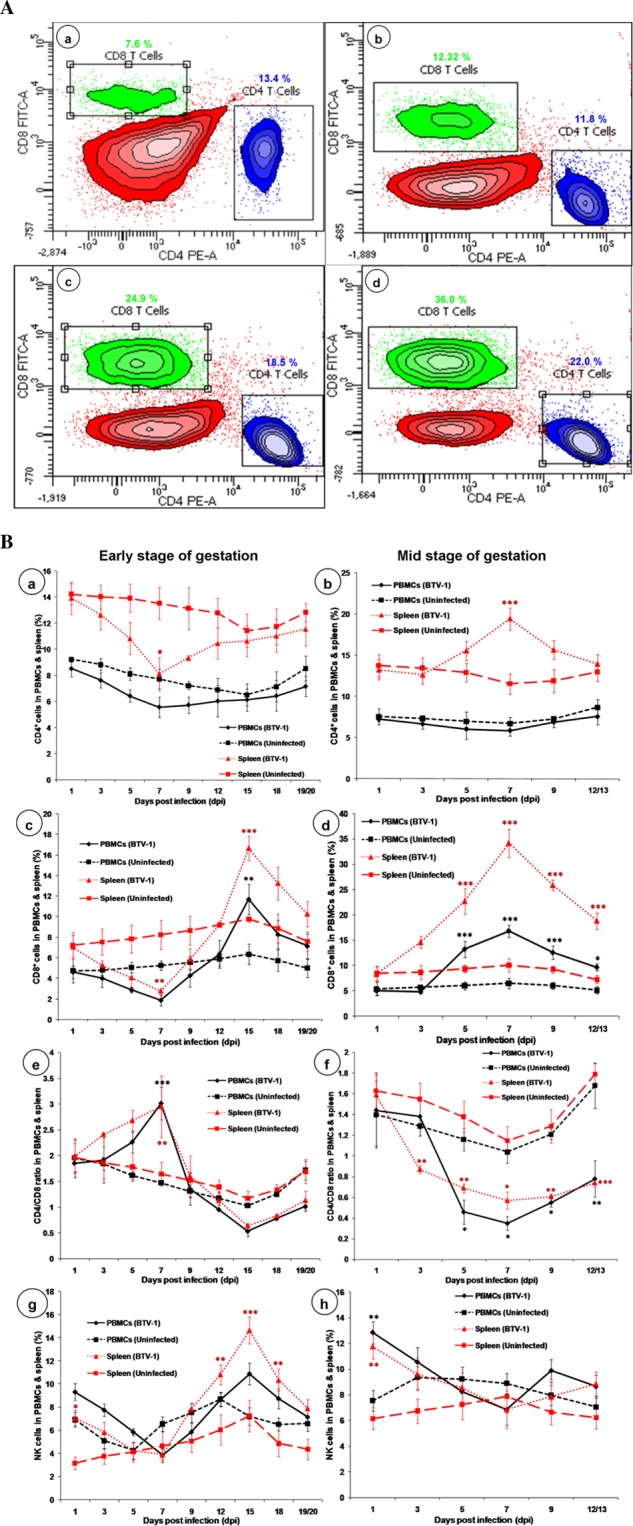
(**A**) Dot-plot showing flow cytometric analysis of CD4^+^ and CD8^+^ T lymphocytes after staining with PE and FITC, respectively in uninfected control (a) and BTV-1 infected groups (b,c,d) during mid stage of gestation at 3 (a,b), 5 (c), and 7 dpi (d). Representative figures of two independent experiments are shown. Lower right quadrant (FL2-H) represents CD4^+^ T lymphocytes (PE), while upper left quadrant (FL1-H) shows CD8^+^ T lymphocytes (FITC). (**B**) Effect of BTV-1 infection on immune cell populations at various time points of pregnant mice infected with BTV-1 during early (a,c,e,g) and mid (b,d,f,h) stages of gestation, and uninfected control group. CD4^+^ T lymphocytes (a,b), CD8+ T lymphocytes (c,d), CD4^+^/CD8^+^ ratio (e,f) and natural killer cells (g,h) in PBMCs (black solid line and square dot line) and spleen (red round dot line and long dash line). Results are presented as line diagram with mean ± SEM at each time point (n = 3). Two-way ANOVA with Bonferroni post-test was used. *P < 0.05, **P < 0.01 and ***P < 0.001 statistically significant compared with uninfected control group.

Analysis of T lymphocyte subsets in spleen and PBMCs of pregnant mice infected with BTV-1 during early stage of gestation revealed significant (P < 0.05) changes at various time points (Fig. 7B). In spleen, CD4^+^ and CD8^+^ T cells were constantly decreased and significant decrease was recorded on 7 dpi. Subsequently, there was increase, but only CD8^+^ T cells showed significant (P < 0.001) peak increase on 15 dpi. In PBMCs, CD4^+^ and CD8^+^ T cells were non-significantly decreased on 7 dpi, and thereafter only CD8^+^ T cells were significantly (P < 0.01) increased on 15 dpi (Fig. 7B).

The kinetics of T lymphocyte subsets in spleen and PBMCs of mid gestation mice revealed significant changes at various time points (Fig. 7A,B). In spleen, CD4^+^ T cells were constantly increased from 3 dpi and significantly peaked on 7 dpi in BTV-1 infected group thereafter, the cells were decreased. In spleen, CD8^+^ T cells constantly increased up to 7 dpi and significant (P < 0.001) increase was observed on 5, 7, 9, and 12/13 dpi (Fig. 7A); however, these cells were decreased after 7 dpi. In PBMCs, CD4^+^ cells showed non-significant changes; however, CD8^+^ T cells significantly (P < 0.001) increased on 5, 7, 9, and 12/13 dpi. In uninfected control group, CD4^+^ and CD8^+^ T cells showed normal physiological fluctuations during pregnancy at same time points (Fig. 7B).

### Kinetics of CD4^+^ and CD8^+^ T cells ratio

The CD4^+^ and CD8^+^ T cells ratio in spleen and PBMCs of early gestation mice revealed significant changes at various time points in BTV-1 infected group (Fig. 7B). In spleen and PBMCs, CD4^+^ and CD8^+^ T cells ratio was significantly increased on 7 dpi, but thereafter, the levels were non-significantly decreased in infected group.

The CD4^+^ and CD8^+^ T cells ratio in spleen and PBMCs of mid gestation mice revealed significant changes at various time points in BTV-1 infected group (Fig. 7B). The CD4^+^ and CD8^+^ T cells ratio was significantly decreased from 3 (spleen) and 5 (PBMCs) dpi onwards in BTV-1 infected group due to significantly increased CD8^+^ T cells. There was no specific trend in CD4^+^ and CD8^+^ T cells ratio noticed in the uninfected control group.

### Kinetics of NK cells in spleen and PBMCs

The kinetics of NK cells in spleen and PBMCs of early gestation mice revealed significant changes at specified time intervals in BTV-1 infected group (Fig. 7B). In spleen, NK cells population significantly (P < 0.05) and in PBMCs, non-significantly increased on 1 dpi. Subsequently, the levels were decreased up to 7 dpi and thereafter, the levels significantly increased on 12, 15, and 18 dpi in spleen and non-significantly increased in PBMCs of BTV-1 infected group. There was no specific trend noticed in NK cells in spleen and PBMCs of uninfected control group.

The kinetics of NK cells in spleen and PBMCs of mid stage pregnant mice, revealed significant (P < 0.01) increase at 1 dpi in BTV-1 infected group (Fig. 7B). Subsequently, the levels were decreased up to 7 dpi and thereafter, the levels increased. There was no specific trend noticed in NK cells in spleen and PBMCs in the uninfected control group.

### Detection of apoptotic cells in PBMCs

The role of apoptosis in BTV-induced pathogenesis during early (Supplementary Fig. S5a–c) and mid stages of gestation by quantifying the annexin V-FITC labelled early apoptotic cells in PBMCs at the specified intervals by flow cytometry was investigated. The percentage of apoptotic cells in PBMCs reached peak levels on 7 dpi (Supplementary Fig. S5) and significantly increased on 5–18 dpi in early and on 3 to 12/13 dpi in BTV-1 infected mid gestation group. There was no specific trend noticed in the percentage of apoptotic cells in PBMCs of uninfected control group (Supplementary Fig. S5).

### *In situ* detection of apoptotic cells in tissues

**Figure 8:**
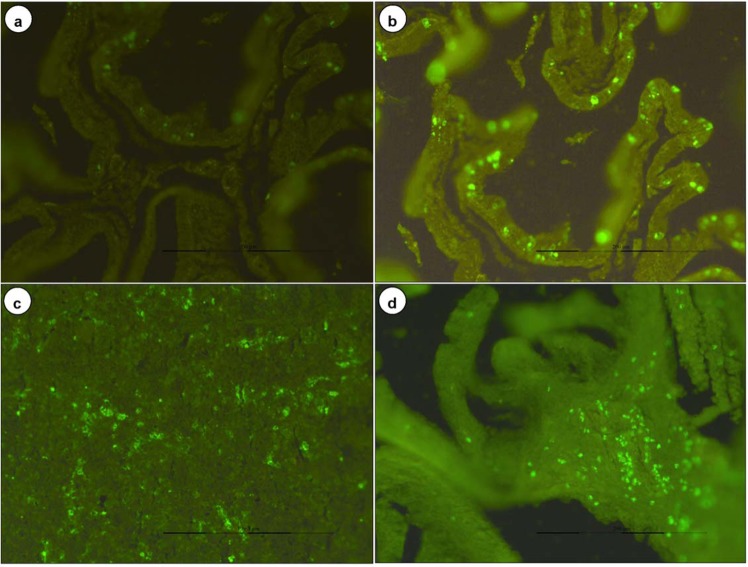
**(a)** Mild green fluorescence signals of apoptotic cells in yolk sac epithelium of placenta in uninfected control mice on 15 dpi. FITC x200. **(b)** Strong green fluorescence signals of apoptotic cells in yolk sac epithelium of placenta in BTV infected mice on 15 dpi. FITC x200. **(c)** Apoptotic cells showed green fluorescence signals in embryo infected with BTV-1 during early stage of gestation. FITC x200. **(d)** BTV-1 infected uterine stromal cells showed strong positive green fluorescence signals of apoptotic cells. FITC x200.

BTV-1 induced apoptotic cell death was detected in formalin fixed and paraffin embedded tissue sections of embryo (early gestation), placenta, uterus, ovary, lymph nodes, spleen, and brain of both groups (Figs. 4e,f, 8a–d). Increased number of BTV-1 induced green fluorescence signals of apoptotic cells were detected on 7 to 9 dpi. Uninfected control animals showed very few positive signals in these organs. Foetuses born from BTV infected mid gestation group showed strong green fluorescence signals of apoptotic cells in brain.

### Alizarin red staining

The effect of BTV-1 in the skeletal system of foetuses born from early and mid stages of gestation at 20/21 GD was studied. The length of different bones in forelimb and hindlimbs, vertebral column (cervical–sacral), and skull (length and diameter) of foetuses born from early and mid stages of gestation showed significant differences (Table 4). The foetuses born from BTV-1 infected mid stage of gestation showed significantly decreased lengths (Supplementary Fig. S6).

**Table 4:** Effect of BTV-1 infection in the skeletal system of foetuses on 20/21 gestation day.

Groups	Fore limb length (mm)				Hind limb length (mm)		Vertebral column length (mm; C7-S4)	Skull (mm)	
	Scapula	Humerus	Radius	Ulna	Femur	Tibia and Fibula		Length	Diameter
Control (Pregnant)	1.82 ± 0.03	1.91 ± 0.05	1.97 ± 0.03	1.43 ± 0.03	1.67 ± 0.03	1.87 ± 0.04	13.44 ± 0.37	6.29 ± 0.21	3.25 ± 0.21
Early stage of gestation	1.77 ± 0.03	1.79 ± 0.04	1.75 ± 0.03*	1.21 ± 0.03	1.43 ± 0.03*	1.72 ± 0.04*	11.78 ± 0.44*	5.56 ± 0.25	4.0 ± 0.35
Mid stage of gestation	1.67 ± 0.03*	1.7 ± 0.04*	1.61 ± 0.03*^,a^	1.09 ± 0.04*^,a^	1.35 ± 0.04*	1.61 ± 0.03*	11.31 ± 0.42*	5.12 ± 0.21*	4.69 ± 0.22*

Values bearing asterisk (*) differs significantly (P ≤ 0.05) in BTV-1 infected group when compared to uninfected control group at specified time intervals (n = 9). ^a^Differs significantly (P ≤ 0.05) in mid infected group when compared to early infected group at specified time intervals. Alizarin red staining was performed to assess the skeletal system.

## Discussion

The present studies perhaps are the first attempts to explore the occurrence of transplacental transmission (TPT) of wild-type Indian BTV-1 in pregnant mice during early (infected on 1 GD) and mid (infected on 8 GD) stages of gestation. Until 2006, BTV serotypes causing TPT were all ascribed to use of modified or cell attenuated vaccine virus strains in cattle^6,28,29^, sheep^30,31^, and dogs^7^. In 2006, BTV-8 in Europe caused significant increase in the incidence of abortions, stillborns, and births of weak or deformed foetuses in sheep, goats, and cattle^2,9^.

The BTV-l used in the present study, was isolated from aborted and stillborn fetal spleen of goats from Sardarkrushinagar, Gujarat, India in 2007^20^. It is postulated that wild type BTV strains might have acquired either cell culture adapted vaccine strain properties resulted in TPT^1,32,33^. Interestingly, TPT in ovine, bovine, and caprine species could be involved in the mechanisms of overwintering and introduction of BTV in free regions as described in Northern Ireland^11^. The serotypes of BTV exist as a swarm of closely related sequence variants with one or several dominant master sequences (quasispecies)^8^. Changes in the environment, vector and host species can lead to selection of sequence variants with an enhanced or reduced virulence for the new environment. These minor viral variants may display phenotypical changes like changes in virulence, tissue tropism, ability to cross the placenta and cause fetal infections^1,34^. However, in contrast, past research findings reveal that natural or experimental infection with wild-type BTV serotypes in pregnant cattle and sheep did not show any vertical or TPT potential^35,36^.

In the present study, BTV-1 infection during early stage of gestation resulted in reduced implantation sites, early embryonic death, abortion, fetal resorption, mummification, and hemorrrages and necrosis of embryos. Exposure of bovine and caprine blastocysts to BTV-8 resulted in active viral replication leads to growth arrest and significant levels of cellular apoptosis^37,38^. This might the reason for lesions noticed during early stage in the present study. The results of the present study have been in concordance with the earlier findings^5,6,10,30^. In Israel and Australia, modified live vaccine strain of BTV-23 in pregnant Merino sheep during 35 to 43 days of gestation caused 40–56% of early foetal mortality with much lesser extent or no CNS malformations like hydranencephaly in their offsprings^31,36^.

The TPT rate differs significantly depending on species of host, stage of gestation, BTV serotype, route of inoculation, immunological status of the host, and dose of virus^32,33^. The present study showed higher rate of TPT during mid stage of gestation (71.43%) when compared to early stage (57.14%). The exact mechanism for gestation stage dependent TPT of BTV is not yet fully elucidated^32,33^. The results of the present study were concorded with the findings of van der Sluijs *et al*.^14^, who reported higher TPT rate (69%) during mid stage of gestation (between 70 and 75 days of gestation) than early stage (40–45 days of gestation) in ewes. van der Sluijs *et al*.^15^ also reported 67% of TPT rate with BTV-1 and 56% with BTV-8 in ewes. Belbis *et al*.^16^ demonstrated 33% of TPT during mid stage of gestation (61 days of pregnancy) but not during late stage (135 days) in goats. The TPT rate in cattle under the field situation varies from 10 to 41.7%^10^. Higher rate of TPT during mid stage of gestation could be due to more intense blood perfusion of the placental endothelium and increased erythrophagocytosis by the embryonal trophoblasts might facilitated the TPT of BTV-1 in the present study^32,33,39^.

In the present study, stomach of BTV-1 infected dam during early stage of gestation contained digested contents of aborted embryos/foetuses due to cannibalization. Cannibalism was due to loss of maternal instinct owing to disturbances in the physical state of the dam. In the present study, BTV-1 induced stress in the dam might be responsible for cannibalistic behaviour. Several reports speculated that cannibalism habit is more common in dams, which delivered malformed or defective or abnormal offspring^40^. This cannibalism effect influences reproductive research in rodents, because female mice would selectively cannibalize their malformed offspring^40^. In the present study, this might be the reason for reporting less number of malformed young ones.

In the present study, identification of BTV from foetuses suggested that viraemic foetuses could introduce infectious BTV into new regions where suitable vectors are active^6,16,32,33^. How long these foetuses remain infectious will be the subject of further investigation. Overwintering mechanisms of BTV still unknown, from an epidemiological point of view, it would be critical to determine whether TPT would facilitate survival of BTV during vector-free period in temperate climates^10,15^. van der Sluijs *et al*.^14,15^ reported the presence of infectious BTV in umbilical cord blood and various foetal tissues with severe pathological consequences. In the present study, positive immunolabelling of BTV-1 antigen was demonstrated in the infiltrated inflammatory cells in mesometrium and embryonic stromal cells in the decidua. Since BTV is associated with monocytes, lymphocytes, red blood cells and endothelial cells, directly facilitates the virus transfer across the placenta along with migration of monocytes/macrophages, and/or by viral transfer through trophoblast cells^7,41–43^. During the initial stages of viremia, there is evidence for the presence of free virus in the plasma. The present study first time demonstrated the BTV-1 localization in the placenta and trophoblast cells by IHC.

In the present study, strong positive immunolabelling of BTV-1 antigen was observed in uterus, placenta, ovary, and CL. De la Concha-Bermejillo *et al*.^29^ demonstrated mild multifocal perivascular cuffing of lymphocytes, several microthrombi in the ovaries and CL of BTV infected heifers, but BTV was neither isolated nor demonstrated by immunofluorescence or *in situ* hybridization in ovaries. However, BTV was isolated from the uterus of heifers. In the present study, presence of BTV antigen in ovary might have responsible for apoptotic changes in ovaries and resulted pregnancy consequences. Presence of high titers of virus in the luteal cells of CL resulted in necrosis of infected cells and loss of pregnancy^29,44^. In the present study, demonstration of BTV from ovaries and CL without much ovarian lesions is similar to the findings of Parsonson *et al*.^44^ BTV could infect luteal cells right after ovulation in viremic animals when high titers of virus are present in blood and the developing stage of corpus hemorrhagicum^29,44^.

In the present study, histopathological lesions in uterus and placenta were concorded with the placental lesions observed in sheep and cattle infected with BTV^14,32,33^. Severe microscopic neurological lesions in the foetuses during mid stage of gestation were concorded with the previous reports^4,15,45^. The congenital anomalies are mainly due to vascular damage and high affinity of BTV for undifferentiated neuronal precursor cells in the brain during development resulting in massive necrosis^4,32,33,45^. In the present study, gross neurological lesions like hydranencephaly or porencephaly in the young ones are not observed. The results were concorded with the previous reports that European strain of BTV-8 during mid stage of gestation (62–75 GD) in goat foetuses did not produce any neurological lesions even after crossing placenta^4,13^. It is well documented that transplacental infection and associated defects can vary widely in sheep and cattle and is therefore unpredictable^7^. In the present study, congestion and haemorrhages were observed in various organs of BTV-1 infected dam, which was concorded with Coetzee *et al*.^13^.

In the present study, BTV-inoculated mice had seroconverted by 7 and 5 dpi and reached peak levels by 15 and 9 dpi in early and mid gestation, respectively. These results were concorded with earlier findings in non-pregnant ewes and goats infected with BTV-8^13,16^. Pregnant ewes were inoculated with either field or rescued strains of BTV-2 and BTV-8 resulted in seroconvertion by 6 dpi and antibody levels reached the diagnostic threshold level by 10 dpi and antibodies levels were maintained up to 28 dpi^46^. The significantly increased antibody levels were well correlated with antigenic stimulation resulted in proliferation of CD4^+^ and CD8^+^ T lymphocytes in spleen and PBMCs, and germinal centers formation with proliferation of B cell areas in lymph nodes and spleen of dams was noticed led to high humoral immune response.

In the present study, BTV-1 infected both groups showed leukopenia and lymphopenia at 7 dpi followed by leukocytosis and lymphocytosis at 15 dpi. Further, CD4^+^ and CD8^+^ T lymphocytes were significantly decreased on 7 dpi and subsequently increased on 15 dpi in spleen and PBMCs. The role of T lymphocyte subsets in the modulation of CMI in pregnant animals infected with BTV-1 has not been studied so far. To our knowledge, this is the first study constituted the detailed characterization of kinetics of haematological parameters and T lymphocyte subsets in spleen and PBMCs in IFNAR1-blocked pregnant mice. CD4 and CD8 T cells work together to eliminate the intracellular pathogens including viruses in association with many secretory molecules^47^.

One of the mechanisms proposed for leukopenia and lymphopenia in sheep was apoptosis^48–50^. Several studies proved that BTV is a potent apoptosis inducer in various mammalian cell lines *in vitro* and played important role in pathogenesis of BT^48–50^. BTV-induced apoptosis happens through both extrinsic (caspase-8 activation) and intrinsic (caspase-9 activation) pathways^48,49^. In the present study, significantly increased apoptosis of PBMCs with significantly decreased TLC and T lymphocyte subsets were observed during peak viraemia on 7 dpi, which was probably due to replication of BTV in lymphocytes, monocytes/macrophages, dendritic cells, and stem cells of haemopoietic system could result in cellular injury and death of cells^43,47,51^. These results were corroborated with the previous studies of experimental infections in non-pregnant sheep, white-tailed deer, and IFNAR^(-/-)^ mice^1,51–53^.

In the present study, leukocytosis and lymphocytosis with significantly increased CD8^+^ T cells was observed on 15 dpi in early gestation, possibly due to antigen presenting cells got the sufficient time for processing and presentation of antigen to CD4 T helper cells, thereby their activation and subsequent proliferation. Lymphocytic-proliferative response indicates CMI response. Similar observation has been made by earlier workers in BTV infected non-pregnant sheep and cattle^47,50,52^. In the present study, CD4^+^ and CD8^+^ T cells ratio was significantly increased on 7 dpi, when CD8^+^ T cells levels are significantly low; however, ratio was low on 15 dpi in BTV-1 infected early gestation group. Other workers also reported similar findings in sheep infected with BTV-1, BTV-8, and BTV-23^47,50,52^.

In the present study, significantly increased CD8^+^ T cells was observed on 7 dpi in BTV infected mid gestation, which resulted in significantly low CD4^+^ and CD8^+^ T cells ratio in PBMCs and spleen. The CD8 cytotoxic T lymphocytes play an important role in killing of virus infected cells by both secretory and membranolytic pathway involving granzyme and perforin, and secreting several antiviral cytokines. CD8^+^ T cells are most important cells during murine pregnancy especially at mid-gestation to protect both mother and foetus from infections by directly recognizing allogeneic MHC class I molecules and regulation to maintain fetal tolerance. Interestingly, viral infections alter the maternal CD8^+^ T cell response by changing the CD8^+^ T cell repertoire and increasing the influx of CD8^+^ T cells to decidual tissue. Usually, high T cell activation threshold at the fetal–maternal interface may prevent efficient clearance of viral infections. But, increased inflammatory response due to viral infections may break fetal–maternal tolerance results in pregnancy complications^54,55^. T cells are highly migratory and their ability to fight infections depends on their tissue localization and their capacity to traffic through different lymphoid and peripheral tissues.

Natural killer (NK) cells are part of innate immune system, plays a key role in host first line of defense against viral infections^56^. In the present study, NK cells increased on 1 dpi in early and mid infection and subsequently decreased on 7 dpi and again increased on later stages of infection. Mouse models provided evidence that NK cells played critical role to control several viral infections like murine cytomegalovirus, poxviruses and influenza virus^56,57^. To our knowledge, this is the first study regarding kinetics of NK cells in spleen and PBMCs in IFNAR1-blocked pregnant mice infected with BTV-1.

In conclusion, present investigation strongly proved the transplacental transmission of wild-type Indian BTV-1 and higher rate of TPT during mid stage than early stage of gestation. During early stage, reduced implantation sites and abortion, and mid stage, neurological lesions in foetuses were noticed. BTV-1 antigen was first time demonstrated in reproductive organs, embryos, and different fetal organs. Transplacentally infected foetuses were viraemic after birth suggested possible source of infection to other animals. Further research remains necessary to investigate the TPT of Indian BTV serotypes in ruminants and to clarify the role of BTV in pre-implantation embryos at a molecular level. The protective efficacy of an inactivated pentavalent vaccine developed for control of BTV in India would be important to investigate against vertical transmission of BTV in pregnant animals. This mice model can also serve to test new vaccines for their efficacy in preventing vertical transmission of BTV.

## Supplementary information

Virological, immunological and pathological findings of transplacentally transmitted bluetongue virus serotype 1 in IFNAR1-blocked mice during early and mid gestation.

## References

[1] MacLachlan NJ, Drew CP, Darpel KE, Worwa G (2009). The pathology and pathogenesis of bluetongue. J. Comp. Pathol..

[2] Toussaint JF (2006). Bluetongue in northern Europe. Vet. Rec..

[3] Darpel KE (2007). Clinical signs and pathology shown by British sheep and cattle infected with bluetongue virus serotype 8 derived from the 2006 outbreak in northern Europe. Vet. Rec..

[4] Osburn BI (1971). Experimental viral-induced congenital encephalopathies. II. The pathogenesis of bluetongue vaccine virus infection in fetal lambs. Lab. Invest..

[5] MacLachlan NJ, Osburn BI (1983). Bluetongue virus induced hydranencephaly in cattle. Vet. Pathol..

[6] MacLachlan NJ, Osburn BI (2008). Induced brain lesions in calves infected with bluetongue virus. Vet. Rec..

[7] Osburn BI (1994). The impact of bluetongue virus on reproduction. Comp. Immunol. Microbiol. Infect. Dis..

[8] Bonneau KR, Mullens BA, MacLachlan NJ (2001). Occurrence of genetic drift and founder effect during quasispecies evolution of the VP2 and NS3/NS3A genes of bluetongue virus upon passage between sheep, cattle, and *Culicoides sonorensis*. J. Virol..

[9] Wilson AJ, Mellor PS (2009). Bluetongue in Europe: past, present and future. Philos. Trans. R. Soc. Lond. B Biol. Sci..

[10] De Clercq K (2008). Transplacental infection and apparently immunotolerance induced by a wild-type bluetongue virus serotype 8 natural infection. Transbound. Emerg. Dis..

[11] Menzies FD (2008). Evidence for transplacental and contact transmission of bluetongue virus in cattle. Vet. Rec..

[12] Batten, C. *et al*. Transplacental transmission of bluetongue serotype 8 (BTV-8) in the United Kingdom (2007–2008). In: Proceedings of the Epizone, 3^rd^ edition, Antalya, Turkey, 12–15 (2009).

[13] Coetzee P (2013). Transplacental infection in goats experimentally infected with a European strain of bluetongue virus serotype 8. Vet. J..

[14] van der Sluijs M (2011). Transplacental transmission of bluetongue virus serotype 8 in ewes in early and mid gestation. Vet. Microbiol..

[15] van der Sluijs MT (2013). Transplacental transmission of bluetongue virus serotype 1 and serotype 8 in sheep: virological and pathological findings. PLoS One.

[16] Belbis G (2013). Evidence of transplacental transmission of bluetongue virus serotype 8 in goats. Vet. Microbiol..

[17] Calvo-Pinilla E, Rodriguez-Calvo T, Anguita J, Sevilla N, Ortego J (2009). Establishment of a bluetongue virus infection model in mice that are deficient in the alpha/beta interferon receptor. PLoS One.

[18] Ortego J, de la Poza F, Marín-López A (2014). Interferon α/β receptor knockout mice as a model to study bluetongue virus infection. Virus Res..

[19] Jameson P, Schoenherr CK, Grossberg SE (1978). Bluetongue virus, an exceptionally potent interferon inducer in mice. Infect. Immun..

[20] Chauhan HC (2014). Isolation of bluetongue virus serotype 1 from aborted goat fetuses. Rev. Sci. Tech..

[21] Sapre SN (1964). An outbreak of bluetongue in goats and sheep. Indian Vet. Rev..

[22] Champlin AK, Dorr DL, Gates AH (1973). Determining the stage of the estrous cycle in the mouse by the appearance of the vagina. Biol. Reprod..

[23] Sheehan KC (2006). Blocking monoclonal antibodies specific for mouse IFN-alpha/beta receptor subunit 1 (IFNAR-1) from mice immunized by *in vivo* hydrodynamic transfection. J. Interferon Cytokine Res..

[24] Smith DR (2017). Neuropathogenesis of Zika virus in a highly susceptible immunocompetent mouse model after antibody blockade of type I interferon. PLoS Negl. Trop. Dis..

[25] Lakshmi IK (2018). Standardization and application of real-time polymerase chain reaction for rapid detection of bluetongue virus. Vet. World.

[26] Madhu BP (2016). Role of nitric oxide in the regulation of immune responses during rabies virus infection in mice. Virusdisease.

[27] Ovchinnikov D. Alcian blue/alizarin red staining of cartilage and bone in mouse. *Cold Spring Harb. Protoc*. **2009**, pdb.prot5170 (2009). 10.1101/pdb.prot5170 20147105

[28] Luedke AJ, Jochim MM, Jones RH (1977). Bluetongue in cattle: Effects of *Culicoides variipennis* transmitted bluetongue virus on pregnant heifers and their calves. Am. J. Vet. Res..

[29] De la Concha-Bermejillo AA, Odeon A, BonDurant RH, Osburn BI (1993). Experimental infection of pregnant cattle with bluetongue virus serotype 11 between postbreeding days 21 and 48. J. Vet. Diagn. Invest..

[30] Shultz G, DeLay P (1955). Losses in newborn lambs associated with bluetongue vaccination of pregnant ewes. J. Am. Vet. Med. Assoc..

[31] Flanagan M, Johnson SJ (1995). The effects of vaccination of Merino ewes with an attenuated Australian bluetongue virus serotype 23 at different stages of gestation. Aust. Vet. J..

[32] Zientara S, Ponsart C (2014). Viral emergence and consequences for reproductive performance in ruminants: two recent examples (bluetongue and Schmallenberg viruses). Reprod. Fertil. Dev..

[33] Maclachlan NJ, Osburn BI (2017). Teratogenic bluetongue and related orbivirus infections in pregnant ruminant livestock: timingand pathogen genetics are critical. Curr. Opin. Virol..

[34] Kirkland, P. D. & Hawkes, R. A. A comparison of laboratory and ‘wild’ strains of bluetongue virus - Is there any difference and does it matter?. *Vet. Ital.* **40**, 448–455 (2004). 20422568

[35] Acree JA (1991). Failure of embryos from bluetongue infected cattle to transmit virus to susceptible recipients or their offspring. Theriogenology.

[36] Shimshony A (2004). Bluetongue in Israel – a brief historical overview. Vet. Ital..

[37] Al Ahmad M.Z. Ali, Pellerin J.L., Larrat M., Chatagnon G., Cécile R., Sailleau C., Zientara S., Fieni F. (2011). Can bluetongue virus (BTV) be transmitted via caprine embryo transfer?. Theriogenology.

[38] Vandaele L (2011). Susceptibility of *in vitro* produced hatched bovine blastocysts to infection with bluetongue virus serotype 8. Vet. Res..

[39] Anderson CK, Jensen R (1969). Pathologic changes in placentas of ewes inoculated with bluetongue virus. Am. J. Vet. Res..

[40] Schardein JL, Petrere JA, Hentz DL, Camp RD, Kurtz SM (1978). Cannibalistic traits observed in rats treated with a teratogen. Lab. Anim..

[41] Whetter LE, Maclachlan NJ, Gebhard DH, Heidner HW, Moore PF (1989). Bluetongue virus infection of bovine monocytes. J. Gen. Virol..

[42] Brewer AW, MacLachlan NJ (1994). The pathogenesis of bluetongue virus infection of bovine blood cells *in vitro*: ultrastructural characterization. Arch. Virol..

[43] Drew CP, Heller MC, Mayo C, Watson JL, Maclachlan NJ (2010). Bluetongue virus infection activates bovine monocyte-derived macrophages and pulmonary artery endothelial cells. Vet. Immunol. Immunopathol..

[44] Parsonson IM (1987). Experimental infection of bulls and cows with bluetongue virus serotype 20. Aust. Vet. J..

[45] Richards WVC, Cordy DR (1967). Bluetongue virus infection: pathologic responses of nervous systems in sheep and mice. Science.

[46] Rasmussen LD (2013). Transplacental transmission of field and rescued strains of BTV-2 and BTV-8 in experimentally infected sheep. Vet. Res..

[47] Ellis JA (1990). T lymphocyte subset alterations following bluetongue virus infection in sheep and cattle. Vet. Immunol. Immunopathol..

[48] Mortola E, Noad R, Roy P (2004). Bluetongue virus outer capsid proteins are sufficient to trigger apoptosis in mammalian cells. J. Virol..

[49] Nagaleekar VK (2007). Bluetongue virus induces apoptosis in cultured mammalian cells by both caspase dependent extrinsic and intrinsic apoptotic pathways. Arch. Virol..

[50] Umeshappa CS, Singh KP, Nanjundappa RH, Pandey AB (2010). Apoptosis and immuno-suppression in sheep infected with bluetongue virus serotype-23. Vet. Microbiol..

[51] McColl KA, Gould AR (1994). Bluetongue virus infection in sheep: haematological changes and detection by polymerase chain reaction. Aust. Vet. J..

[52] Sánchez-Cordón PJ (2015). Comparative analysis of cellular immune responses and cytokine levels in sheep experimentally infected with bluetongue virus serotype 1 and 8. Vet. Microbiol..

[53] Marín-López A (2016). Pathological characterization of IFNAR^(−/−)^ mice infected with bluetongue virus serotype 4. Int. J. Biol. Sci..

[54] Constantin CM (2007). Normal establishment of virus-specific memory CD8 T cell pool following primary infection during pregnancy. J. Immunol..

[55] Tilburgs T, Strominger JL (2013). CD8^+^ effector T cells at the fetal-maternal interface, balancing fetal tolerance and antiviral immunity. Am. J. Reprod. Immunol..

[56] Paolini R, Bernardini G, Molfetta R, Santoni A (2015). NK cells and interferons. Cytokine Growth Factor Rev..

[57] Bukowski JF, Woda BA, Habu S, Okumura K, Welsh RM (1983). Natural killer cell depletion enhances virus synthesis and virus-induced hepatitis *in vivo*. J. Immunol..

