# Supplementary material for: Virological, immunological and pathological findings of transplacentally transmitted bluetongue virus serotype 1 in IFNAR1-blocked mice during early and mid gestation
Source: Sci Rep. 2020 Feb 7;10:2164. doi: 10.1038/s41598-020-58268-0 (PMC7005837; doi:10.1038/s41598-020-58268-0)
Supplement: Supplementary file 1 — Virological, immunological and pathological findings of transplacentally transmitted bluetongue virus serotype 1 in IFNAR1-blocked mice during early and mid gestation. [file 41598_2020_58268_MOESM1_ESM.docx]

**Virological, immunological and pathological findings of transplacentally transmitted bluetongue virus serotype 1 in IFNAR1-blocked mice during early and mid gestation**

M. Saminathan^1^, K.P. Singh^2*^, Vineetha S^1^, Madhulina Maity^1^, S.K. Biswas^3^, G.B. Manjunathareddy^4^, H.C. Chauhan^5^, A.A.P. Milton^6^, M.A. Ramakrishnan^3^, Sushila Maan^7^, N.S. Maan^7^, D. Hemadri^4^, B.S. Chandel^5^, V.K. Gupta^2^ and P.P.C. Mertens^8^

^1^Division of Pathology, ^2^Centre for Animal Disease Research and Diagnosis (CADRAD), ICAR-Indian Veterinary Research Institute (ICAR-IVRI), Izatnagar, Bareilly-243122, Uttar Pradesh; ^3^Division of Virology, ICAR-IVRI, Mukteswar Campus, Nainital-263138, Uttarakhand; **^4^ICAR-National Institute of Veterinary Epidemiology and Disease Informatics, Bengaluru-560064, Karnataka; ^5^Department of Veterinary Microbiology, College of Veterinary Science and Animal Husbandry, Sardarkrushinagar Dantiwada Agricultural University, Sardarkrushinagar-385506, Gujarat;** ^6^Division of Animal Health, ICAR-RC for NEH Region, Umiam, Barapani-793103, Meghalaya; ^7^College of Veterinary Sciences, LLR University of Veterinary and Animal Sciences, Hisar- 125 004, Haryana, India; ^8^School of Veterinary Medicine and Science, University of Nottingham, Sutton Bonnington, Leicestershire, UK.

***Corresponding author:** Dr Karam Pal Singh, Principal Scientist, CADRAD, ICAR-IVRI, Izatnagar, Bareilly, Uttar Pradesh, India. e-mail: karam.singh@rediffmail.com; TeleFax: 0091-581-2302188.

**Supplementary Tables**

**Table S1. Effect of BTV-1 infection on early stage of gestation (n = 3 dams at each time point)**

| **Groups** | **Days post infection (dpi)** | | | | | | |
| --- | --- | --- | --- | --- | --- | --- | --- |
|  | **5 (6 GD)** | **7 (8 GD)** | **9 (10 GD)** | **12 (13 GD)** | **15 (16 GD)** | **18 (19 GD)** | **19/20 (20/21 GD)** |
| **No. of implantation sites in both uterine horns** | | | | | | | |
| **Control** | 9.33±0.33 | 10.33±0.33 | 9.67±0.33 | 9.0±0.58 | 10.33±0.33 | 9.67±0.33 | 10.0±0.58 |
| **BTV-1** | 7.0±0.58** | 8.33±0.33** | 6.33±0.33*** | 7.67±0.33 | 6.67±0.33*** | 7.67±0.33** | 8.33±0.33* |
| **No. of live embryos (up to 14 GD) or foetuses (15 to 21 GD)** | | | | | | | |
| **Control** | 9.33±0.33 | 10.33±0.33 | 9.67±0.33 | 9.0±0.58 | 10.0±0.0 | 9.67±0.33 | 10.0±0.58 |
| **BTV-1** | 5.33±0.33*** | 5.0±0.58*** | 3.67±0.33*** | 4.67±0.33*** | 5.33±0.33*** | 5.67±0.33*** | 6.67±0.67*** |
| **No. of dead embryos (up to 14 GD) or foetuses (15 to 21 GD)** | | | | | | | |
| **Control** | Nil | Nil | Nil | Nil | 0.33±0.33 | Nil | Nil |
| **BTV-1** | 1.67±0.33 | 3.33±0.33 | 2.67±0.33 | 3.0±0.58 | 1.33±0.67 | 2.0±0.58 | 1.67±0.88 |

Values bearing asterisk (***)** differs significantly in BTV-1 infected group when compared to uninfected control group at specified time intervals. *P < 0.05; **P<0.01; ***P<0.001

**Table S2. Effect of BTV-1 infection on mid stage of gestation (n = 3 dams at each time point)**

| **Groups** | **Days post infection (dpi)** | | | | | |
| --- | --- | --- | --- | --- | --- | --- |
|  | **1 (9 GD)** | **3 (11 GD)** | **5 (13 GD)** | **7 (15 GD)** | **9 (17 GD)** | **12/13 (20/21 GD)** |
| **No. of implantation sites in both uterine horns** | | | | | | |
| **Control** | 9.33±0.33 | 10.33±0.33 | 10.0±0.58 | 9.67±0.33 | 10.33±0.33 | 9.67±0.33 |
| **BTV-1** | 9.0±0.58 | 9.67±0.33 | 9.67±0.33 | 9.67±0.33 | 10.0±0.58 | 9.67±0.33 |
| **No. of live embryos (up to 14 GD) or foetuses (15 to 21 GD)** | | | | | | |
| **Control** | 9.33±0.33 | 10.33±0.33 | 10.0±0.58 | 9.67±0.33 | 10.33±0.33 | 9.67±0.33 |
| **BTV-1** | 9.0±0.58 | 9.67±0.33 | 8.67±0.33 | 7.67±0.33* | 9.0±0.58 | 8.67±0.33 |
| **No. of reabsorbed embryos/foetuses** | | | | | | |
| **Control** | Nil | Nil | Nil | Nil | Nil | Nil |
| **BTV-1** | Nil | Nil | 1.0±0.0 | 2.0±0.0 | 1.0±0.58 | 1.0±0.0 |

Values bearing asterisk (***)** differs significantly (P≤0.05) in BTV-1 infected group when compared to uninfected control group at specified time intervals.

**Supplementary Figures**


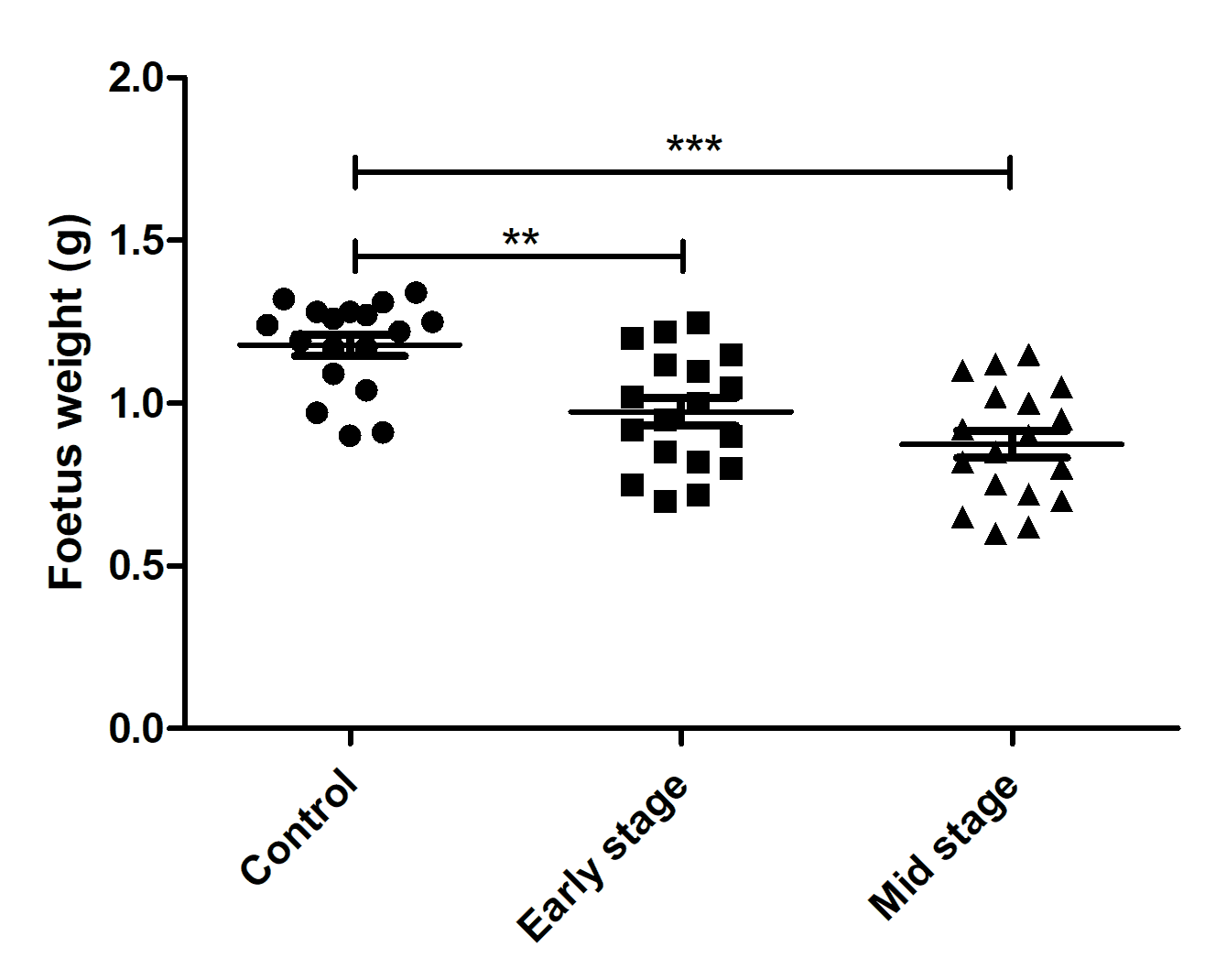


**Figure S1.** Body weight of foetuses born from dams infected with BTV during early (infected on 1 GD) and mid (8 GD) stages of gestation, and uninfected control mice sacrificed on 20/21 GD. Eighteen foetuses from three separate mothers from each group (n = 18 per group). One-way ANOVA with Tukey’s post-test was used, and data were expressed as median. **P<0.01 and ***P<0.001 statistically significant compared with uninfected control group.


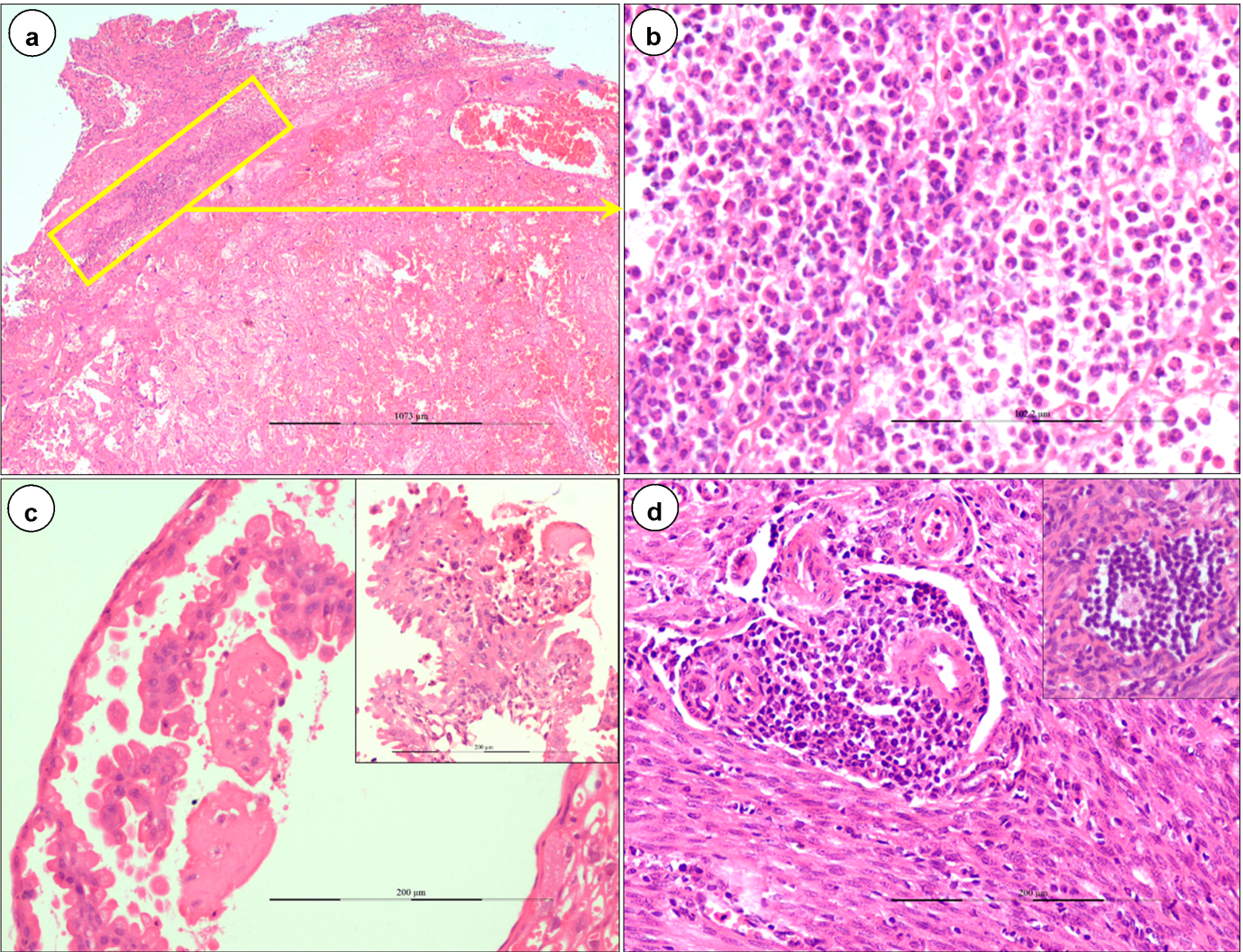


**Figure S2. Histopathological lesions in BTV-1 infected mid gestation group. (a)** Necrosis of metrial gland area, infiltration of inflammatory cells, severe congestion and dilatation of blood vessels in the placenta on 9 dpi. H&E x40. **(b)** Higher magnification showed necrotic areas severely infiltrated with neutrophils, macrophages, and few plasma cells and lymphocytes. H&E x400. **(c)** Fusion and necrosis of yolk sac epithelium of placenta and inflammatory cell infiltration in lamina propria on 9 dpi. H&E x200. **(d)** Marked infiltration of lymphocytes in perivasular areas and myometrium of uterus on 7 dpi. H&E x200.


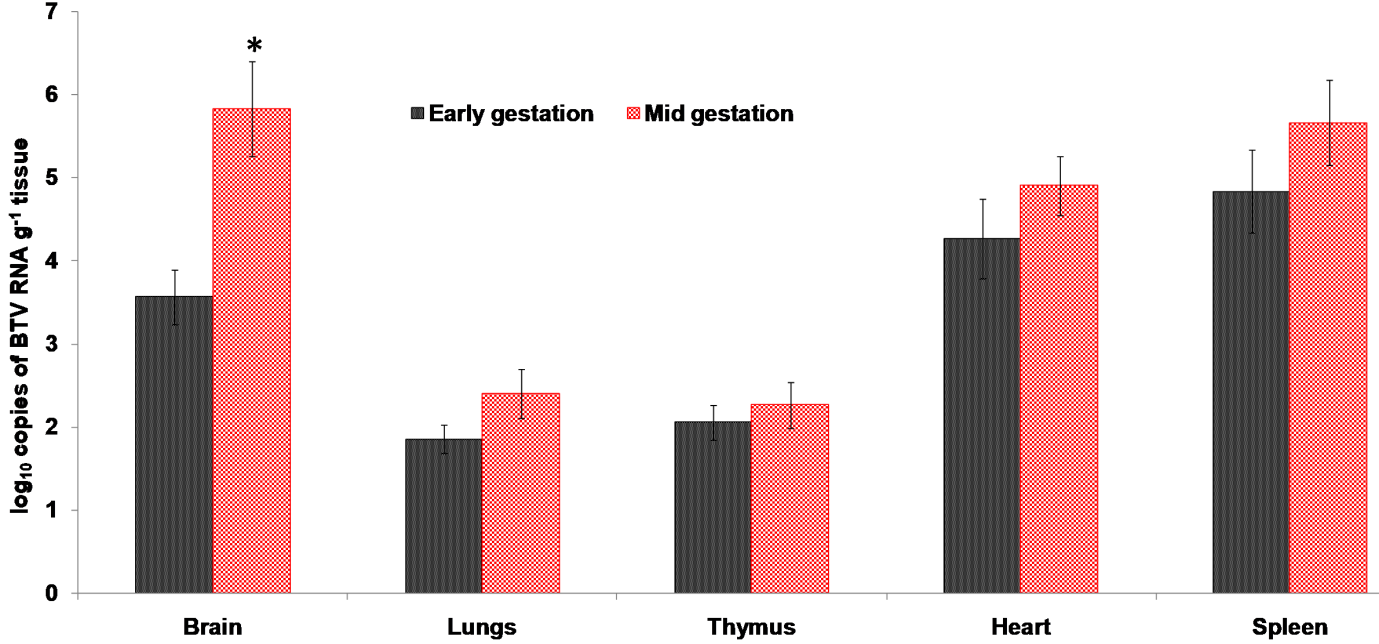


**Figure S3.** Quantification of BTV RNA from foetal tissues born from dams infected with BTV during early and mid stages of gestation on 20/21 GD using TaqMan probe-based real-time PCR assay. Results are presented as bar diagram with mean±SEM at each time point. Six foetuses from three separate mothers from each group (n = 6 per group). Two-tailed unpaired t test was used. *P<0.05 statistically significant compared with early stage of gestation. Virus load in tissues is given as log_10_ copies of viral RNA g^-1^ tissue.

**
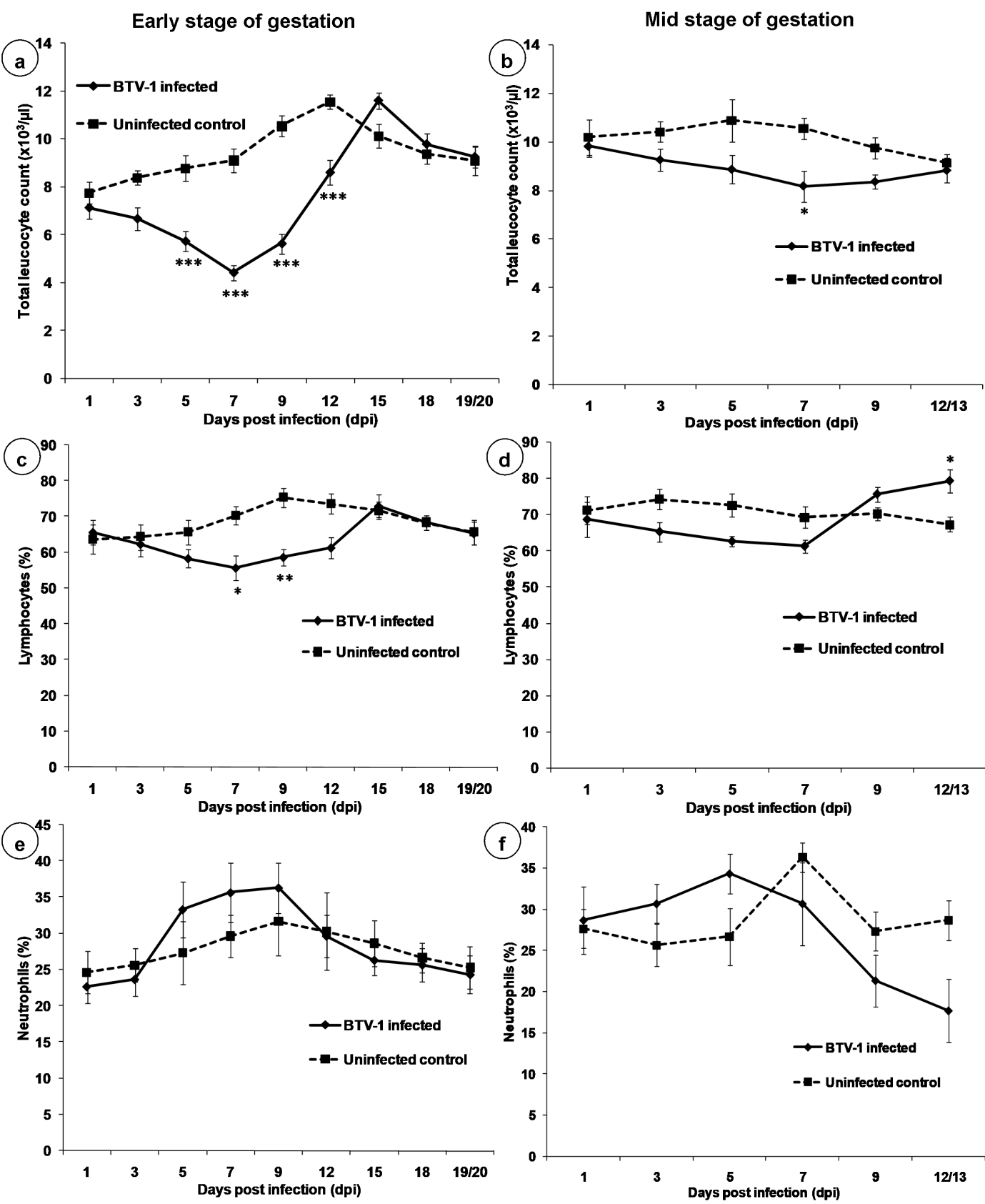
**

**Figure S4.** Effect of BTV-1 infection on haematological values at various time points of pregnant mice infected with BTV-1 (solid line) during early and mid stages of gestation, and uninfected control group (square dotted line). Total leukocyte count (x10^3^/µl), lymphocytes (%), and neutrophils (%) in blood samples from early **(a,c,e)** and mid **(b,d,f)** stages of pregnant mice. Results are presented as line diagram with mean±SEM at each time point (n = 3). Two-way ANOVA with Bonferroni post-test was used. *P<0.05, **P<0.01 and ***P<0.001 statistically significant compared with uninfected control group.


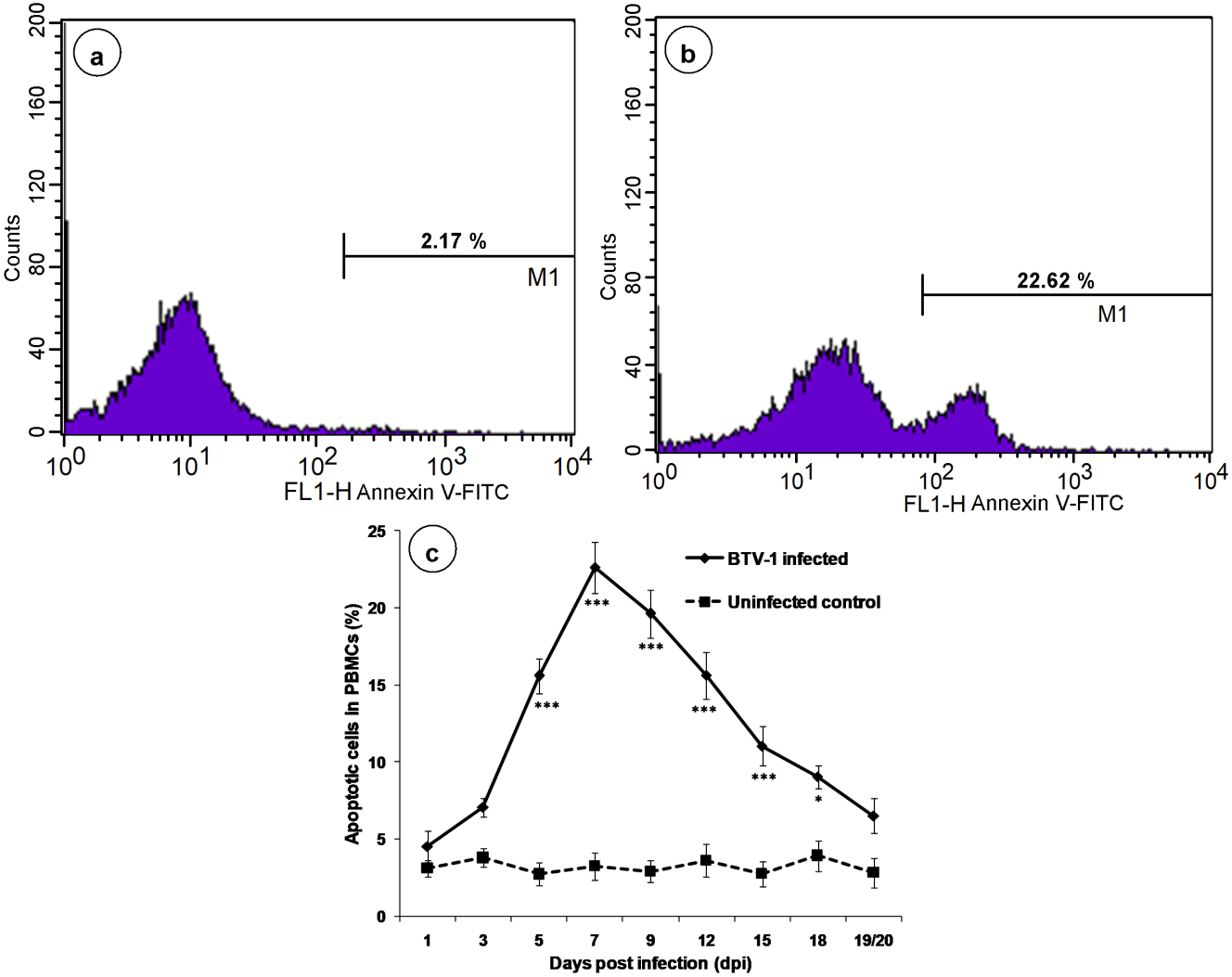


**Figure S5.** Representative histogram showing the flow cytometric analysis of Annexin V-FITC staining in uninfected control **(a)** and BTV-1 infected pregnant mice **(b)** during early stage of gestation at 7 dpi. Marker M1 in the histogram represents the percentage of early apoptotic cells population. **(c)** Line diagram showing the percentage of early apoptotic cells in uninfected control (square dotted line) and BTV-1 infected pregnant mice (solid line). Results are presented as mean±SEM at each time point (n = 3). Two-way ANOVA with Bonferroni post-test was used. *P<0.05 and ***P<0.001 statistically significant compared with uninfected control group.


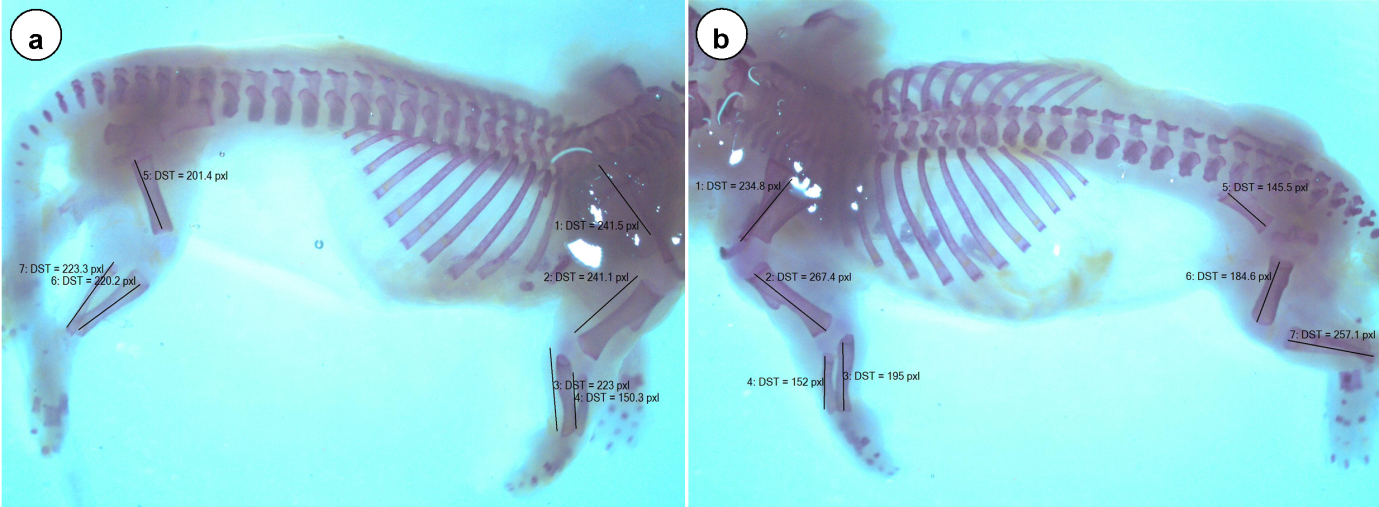


**Figure S6.** Lateral view of foetuses born from dams infected with BTV-1 during mid (8 GD) stage of gestation **(B)** and uninfected control mice **(A)** sacrificed on 20/21 GD stained with Alizarin red.
